# 1,8-Naphthalimide: A Versatile Platform for the Design of Fluorescent Probes Targeting Hydrogen Sulfide in Biological Systems

**DOI:** 10.3390/molecules31173126

**Published:** 2026-09-07

**Authors:** Riley Grieser, Sara Fox-Belmonte, Hector Palencia, Haishi Cao

**Affiliations:** Department of Chemistry, University of Nebraska at Kearney, 2504 9th Ave, Kearney, NE 68849, USA

**Keywords:** 1,8-naphthalimide, hydrogen sulfide, H_2_S, reaction-based probe, fluorescent probe, H_2_S recognition, H_2_S sensor

## Abstract

Hydrogen sulfide (H_2_S) has emerged as an important gaseous signaling molecule involved in numerous physiological and pathological processes, including redox regulation, inflammation, and cellular signaling. Accurate detection of H_2_S in biological systems is therefore essential for understanding its roles in health and disease. Among various fluorescent platforms, the 1,8-naphthalimide scaffold has attracted considerable attention due to its unique photophysical properties, structural tunability, and high sensitivity to substituent modification. This review summarizes recent advances in the development of 1,8-naphthalimide-based fluorescent probes for H_2_S detection. The fundamental photophysical mechanisms associated with the 1,8-naphthalimide fluorophore, including intramolecular charge transfer (ICT), photoinduced electron transfer (PET), and Förster resonance energy transfer (FRET), are discussed in relation to probe design. Emphasis is placed on reaction-based sensing strategies, highlighting organic reactions that enable selective recognition of H_2_S, such as reduction, nucleophilic addition, nucleophilic substitution, and intramolecular cyclization. Representative probes are summarized and compared with respect to sensing mechanisms and photophysical behavior. In addition, the current challenges of 1,8-naphthalimide-based H_2_S probes are discussed. We hope this review will provide useful perspectives for the development of efficient 1,8-naphthalimide-based fluorescent probes for H_2_S detection.

## 1. Introduction

Hydrogen sulfide (H_2_S), once regarded solely as a toxic environmental pollutant, is now recognized as an endogenous signaling molecule of significant physiological relevance in mammals [1]. Together with nitric oxide (NO) and carbon monoxide (CO), H_2_S is classified as a gasotransmitter, a group of small gaseous mediators that readily diffuse across membranes and regulate diverse biological processes [2]. Endogenous H_2_S is generated primarily from L-cysteine through enzymatic reactions catalyzed by cystathionine β-synthase (CBS, EC 4.2.1.22), cystathionine γ-lyase (CSE, EC 4.4.1.1), and 3-mercaptopyruvate sulfurtransferase (3-MST, EC 2.8.1.2) [3]. These enzymes display distinct tissue and subcellular distributions: CBS is predominantly expressed in the central nervous system, CSE is abundant in the cardiovascular system, and 3-MST is localized mainly in mitochondria [4,5,6]. Such spatial organization enables precise, context-dependent regulation of H_2_S biosynthesis across tissues and intracellular compartments [7].

The concentration of free H_2_S in mammalian plasma remains difficult to accurately determine because H_2_S exists in dynamic equilibrium with HS^−^ and can be rapidly consumed, oxidized, or incorporated into bound sulfur pools. Recent studies suggest that the concentration of free H_2_S is approximately 0.7–3 μM, substantially lower than the 10–300 μM concentrations reported in earlier studies [8]. At physiological pH, H_2_S is present predominantly as hydrosulfide (HS^−^, ~80%), a nucleophilic and redox-active species capable of interacting with protein thiols, metal centers, and oxidized biomolecules [9,10]. These intrinsic chemical properties underpin the diverse biological functions of H_2_S [11]. Functionally, H_2_S plays a central role in maintaining cellular homeostasis [12]. A principal mechanism of action involves post-translational modification of cysteine residues via persulfidation (S-sulfhydration), which modulates protein structure, enzymatic activity, and downstream signaling pathways [13]. Through such mechanisms, H_2_S participates in the regulation of antioxidant defense, inflammation, vascular tone, neuronal signaling, and cellular metabolism [14].

In redox regulation, H_2_S enhances cellular antioxidant capacity by promoting glutathione biosynthesis and activating cytoprotective pathways, including the Nrf2 signaling cascade [15,16]. Reduced endogenous H_2_S levels are frequently associated with elevated oxidative stress and increased cellular damage [17]. H_2_S also modulates inflammatory signaling. In many experimental contexts, it suppresses pro-inflammatory pathways and attenuates inflammatory cytokine production [18]. Conversely, dysregulation of H_2_S metabolism may exacerbate inflammatory responses and contribute to chronic disease progression [19]. In this context, mitochondria play a central role in H_2_S biology, functioning as both a source and a target of H_2_S [20]. At low concentrations, H_2_S can serve as an electron donor to the mitochondrial respiratory chain, thereby supporting ATP generation and maintaining bioenergetic homeostasis [21]. In contrast, elevated H_2_S concentrations inhibit cytochrome c oxidase (complex IV), impair oxidative phosphorylation, and may induce mitochondrial dysfunction and cell death [22]. These dual effects highlight the concentration-dependent nature of H_2_S signaling and underscore the necessity for tight spatiotemporal regulation of intracellular H_2_S levels [23]. Aberrant H_2_S metabolism has been implicated in a broad spectrum of pathological conditions, including cardiovascular diseases, metabolic disorders, neurodegenerative diseases, and cancer [24,25,26,27,28]. For example, diminished H_2_S production is associated with endothelial dysfunction and hypertension, whereas certain tumors exhibit upregulated expression of H_2_S-producing enzymes, potentially conferring growth and survival advantages [29]. Together, these observations highlight the context-dependent roles of H_2_S in both physiological and pathological processes.

Despite significant advances in understanding H_2_S biology, accurate quantification of this gasotransmitter in living systems remains challenging [30]. The volatility, rapid metabolism, and susceptibility of H_2_S to oxidation complicate its reliable detection. Conventional analytical methods include colorimetric assays, high-performance liquid chromatography (HPLC), gas chromatography (GC), mass spectrometry, and electrochemical techniques [31,32,33,34,35]. Although these approaches provide direct and quantitative measurements of H_2_S in biological samples, they have inherent limitations, particularly in complex biospecimens. Importantly, most of them are not well suited for real-time, in situ monitoring in living systems, which is essential for establishing quantitative correlations between H_2_S dynamics and biological outcomes [36]. Furthermore, extensive sample preparation and reliance on specialized instrumentation limit their broader application in biological research and clinical settings, especially for dynamic measurements [37].

Fluorescent sensing offers significant advantages for the detection of hydrogen sulfide (H_2_S), particularly in biological environments where high sensitivity and real-time monitoring are essential [38]. Due to their high signal-to-noise ratios and intrinsic signal amplification, fluorescence-based methods enable the detection of H_2_S at low, physiologically relevant concentrations [39]. In contrast to traditional analytical techniques, fluorescent probes allow noninvasive, real-time visualization of H_2_S dynamics in living cells and tissues, providing valuable spatiotemporal information about its production and distribution [40]. Moreover, rational molecular design permits the incorporation of H_2_S-responsive recognition motifs, thereby affording high selectivity over other reactive sulfur, oxygen, and nitrogen species [41]. Fluorescent probes are also compatible with advanced imaging techniques, including confocal and two-photon microscopy, facilitating subcellular localization and quantitative analysis [42]. Collectively, these features make fluorescent sensing a powerful and versatile approach for elucidating the complex biological roles of H_2_S.

1,8-Naphthalimide is a well-established scaffold that has attracted sustained attention in dye chemistry and biomedical research [43]. As a fluorophore, it has been widely employed in the development of fluorescent dyes, optical brighteners, and imaging agents due to its favorable photostability and synthetic versatility [44]. Beyond imaging, certain 1,8-naphthalimide derivatives have demonstrated notable biological activities, including anticancer properties, with several analogues investigated as DNA intercalators or topoisomerase inhibitors [45]. Although these therapeutic applications highlight the pharmacological relevance of the scaffold, its most prominent impact in chemical biology arises from its distinctive photophysical characteristics and modular structural tunability.

Structurally, 1,8-naphthalimide consists of a rigid, planar naphthalene core fused with an imide functionality, forming a highly conjugated π-system. This architecture confers efficient fluorescence emission with relatively high quantum yields [46]. The substituents on the naphthalene ring can significantly modulate the electron density distribution across the π-system, thereby altering absorption and emission wavelengths, fluorescence intensity, and solvatochromic behavior. As a result, subtle structural modifications often lead to substantial changes in photophysical properties, including bathochromic shifts, enhanced brightness, or environment-sensitive emission [47]. In addition, the location of substituents on the naphthalene ring, such as at C3 and C4 positions, can also significantly alter the fluorescence properties [48,49]. This strong structure–property relationship makes 1,8-naphthalimide a privileged platform for the rational design of fluorescent probes.

Importantly, the substituents appended to the naphthalene moiety can serve dual roles: tuning fluorescence and acting as reactive recognition sites [50]. In the context of hydrogen sulfide (H_2_S) detection, functional groups appended on the 1,8-naphthalimide framework-such as azides, nitro groups, and dinitrophenyl ethers can undergo specific chemical transformations upon reaction with H_2_S [51]. These reactions often restore or enhance electron-donating ability, thereby producing a measurable fluorescence change and resulting in a fluorescence “turn-on” response. Such reaction-based sensing strategies enable selective and quantitative detection of H_2_S, as the fluorescence output is directly coupled to a well-defined chemical transformation [52]. The inherent responsiveness of substituents on the 1,8-naphthalimide core thus provides a robust mechanistic foundation for probe development.

In addition to electronic tunability, the *N*-imide moiety offers a versatile handle for modulating physicochemical properties. Substitution at this position does not significantly disrupt the conjugated chromophore but can dramatically influence solubility, polarity, and overall molecular stability. By introducing hydrophilic groups, such as short polyethylene glycol chains or charged functionalities, water solubility and biocompatibility can be enhanced. Conversely, the incorporation of more hydrophobic substituents can facilitate membrane permeability [53]. Through judicious design at the *N*-imide, 1,8-naphthalimide-based probes can be optimized for efficient cellular uptake and minimal cytotoxicity, ensuring compatibility with live-cell imaging applications.

Furthermore, the *N*-imide substituent can be engineered to incorporate targeting motifs that direct the probe to specific subcellular organelles. For instance, attachment of morpholine groups can promote lysosomal localization, while pyridinium units can facilitate mitochondrial targeting [54]. Other peptide or small-molecule ligands may guide probes to organelles such as ribosomes or other intracellular compartments [55]. This modularity enables spatially resolved detection of H_2_S within defined cellular microenvironments, thereby providing insights into localized signaling events and organelle-specific redox regulation. Collectively, the combination of a photophysically robust chromophore, pronounced structure–property tunability, reactive substituent chemistry, and adaptable *N*-imide functionalization establishes 1,8-naphthalimide as an exceptionally versatile fluorophore. These attributes make it an ideal molecular platform for the rational design of fluorescent probes capable of selective, sensitive, and spatially resolved detection of H_2_S in complex biological systems.

## 2. Mechanisms Used for H_2_S Sensing

### 2.1. Intramolecular Charge Transfer (ICT)

**Figure 1:**
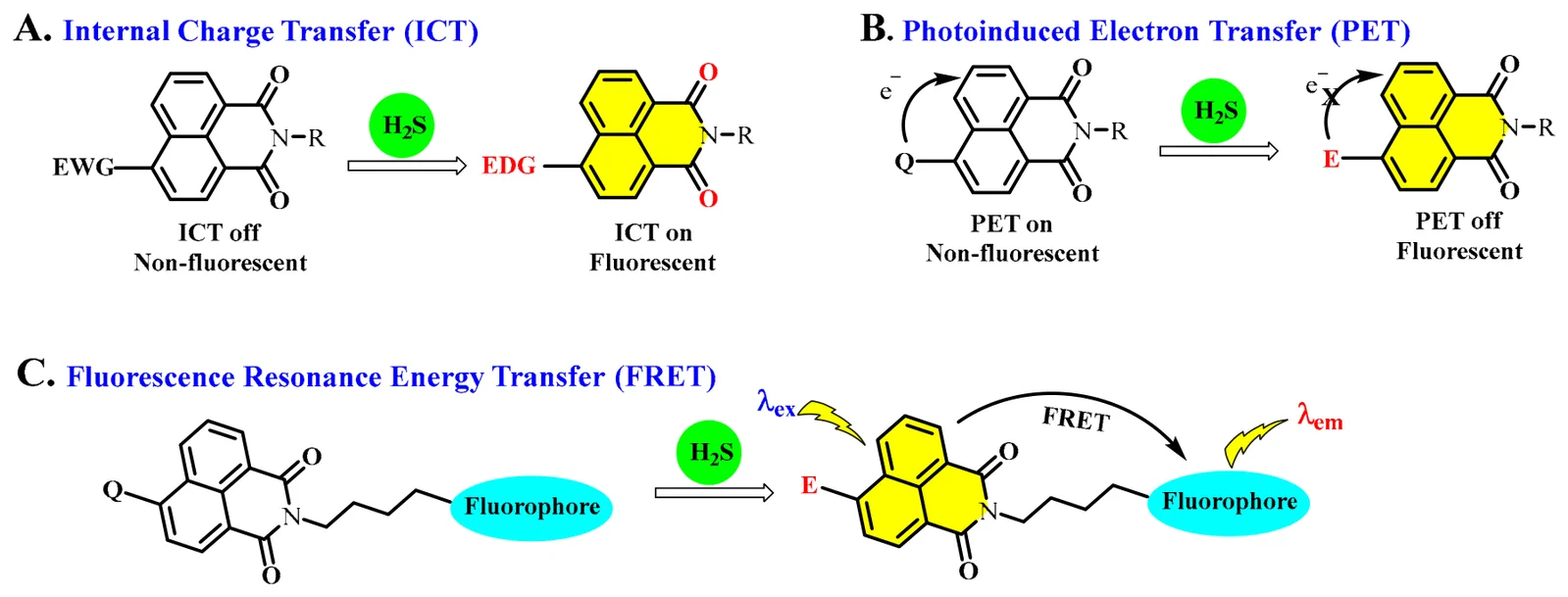
The fluorescence sensing mechanisms for detecting H_2_S based on the 1,8-naphthalimide scaffold.

ICT is a key photophysical characteristic of 1,8-naphthalimide and has been widely exploited for the quantitative detection of H_2_S. The 1,8-naphthalimide scaffold features an electron-deficient imide group conjugated to a π-extended aromatic system. Introduction of electron-donating substituents, typically at the 4-position, generates a donor–π–acceptor framework. Upon photoexcitation, electron density shifts from the donor toward the imide acceptor, producing a charge-separated excited state characteristic of ICT transitions [56]. The strength of ICT process depends on the electronic nature of the substituents. Stronger electron donating groups enhance charge-transfer character, often resulting in bathochromic shifts in both absorption and emission spectra. In addition, ICT states are highly sensitive to environmental factors such as solvent polarity and local dielectric constant, leading to solvatochromic fluorescence behavior [57]. Importantly, H_2_S-triggered chemical reactions that change electron-withdrawing substituents into electron-donating groups can significantly alter ICT efficiency, producing measurable fluorescence “turn-on” response [58]. This predictable modulation of ICT forms the mechanistic basis for designing of 1,8-naphthalimide-based fluorescent probes for the detection of H_2_S (Figure 1A).

### 2.2. Photoinduced Electron Transfer (PET)

Photoinduced electron transfer (PET) is a photophysical process in which light absorption generates an excited electronic state that enables electron transfer between a fluorophore and a nearby donor or acceptor moiety [59]. Upon excitation, the fluorophore becomes either a stronger oxidant or reductant relative to its ground state, creating a thermodynamic driving force for intramolecular electron transfer [60]. Electron-transfer introduces a nonradiative decay pathway that competes with fluorescence emission, typically resulting in signal quenching [61]. The efficiency of PET is governed by the relative redox potentials of the donor–acceptor pair, their electronic coupling, and the spatial separation between them [62].

Within the 1,8-naphthalimide platform, PET is typically implemented through the incorporation of an electron-rich substituent at the 4-position, or less commonly at the 3-position (Figure 1B). These substitution sites provide effective electronic communication with the π-conjugated fluorophore, thereby modulating its excited-state redox properties [63,64]. In many reported systems, a secondary or tertiary amine is introduced as an electron-donating unit. Upon photoexcitation, the naphthalimide excited state exhibits increased oxidizing character, which facilitates intramolecular electron transfer from the appended amine to the fluorophore. formation of the resulting charge-separated state promotes nonradiative decay, leading to efficient fluorescence quenching [65,66].

This mechanistic framework has been widely exploited in the development of H_2_S-responsive fluorescent probes [67]. Among the various recognition motifs, the 2,4-dinitrobenzenesulfonyl (DNBS) ester is frequently employed as an effective fluorescence quencher and reactive trigger. When covalently installed onto the 1,8-naphthalimide scaffold, the DNBS group attenuates fluorescence through PET-mediated quenching. In the presence of H_2_S, selective cleavage of the DNBS moiety occurs via nucleophilic aromatic substitution (S_N_Ar), leading to removal of the sulfonyl group. This transformation substantially alters the electronic properties of the substituent, thereby suppressing the PET process and restoring fluorescence with a characteristic “off–on” response. Such analyte-induced modulation of PET provides a well-defined chemical basis for signal transduction, enabling sensitive and selective fluorescence detection of H_2_S in complex biological systems [68].

### 2.3. Fluorescence Resonance Energy Transfer (FRET)

Fluorescence (Förster) resonance energy transfer (FRET) is a nonradiative energy transfer process in which excitation energy is transferred from a donor fluorophore to a proximal acceptor chromophore via long-range dipole–dipole coupling, providing a powerful spectroscopic ruler for distances in the 1–10 nm range [69]. The transfer efficiency depends on the Förster distance (R_0_), which reflects spectral overlap between donor emission and acceptor absorption (Figure 1C).

1,8-Naphthalimide is an attractive fluorophore scaffold for FRET-based probe design because of its favorable photophysical properties, synthetic tunability, and compatibility with biological systems. It typically exhibits high fluorescence quantum yield, strong absorption in the UV–visible region, and good photostability, making it a reliable donor or acceptor component in FRET constructs [70]. Importantly, the 1,8-naphthalimide core is highly modular: substitutions at the 4-position and imide nitrogen allow precise tuning of electron-donating or -withdrawing character, which in turn modulates emission wavelength, Stokes shift, and charge-transfer character [71]. This tunability facilitates optimization of spectral overlap with a chosen FRET partner and enables rational adjustment of the Förster distance [72]. Moreover, ICT and PET mechanisms can be integrated with FRET to produce amplified or dual-mode responses [73]. Collectively, these features make 1,8-naphthalimide a versatile and rational platform for constructing sensitive, ratiometric FRET probes.

In addition, FRET offers significant advantages for the detection of H_2_S in biological systems due to its capacity for ratiometric signal output [74]. H_2_S-responsive FRET probes are typically designed such that a specific chemical reaction modulates the donor–acceptor coupling by altering spectral overlap. This reaction-induced change produces a measurable shift in FRET efficiency, enabling quantitative detection. A principal advantage is the ratiometric readout, which minimizes artifacts arising from probe concentration, photobleaching, and excitation intensity fluctuations [75]. This feature is particularly important for H_2_S, a reactive and diffusible gasotransmitter present at low and spatially heterogeneous concentrations [76]. FRET-based probes also allow monitoring of endogenous H_2_S generation in live cells with improved signal-to-background ratios compared to single-fluorophore turn-on probes [77]. When combined with fluorescence lifetime imaging, FRET platforms provide robust, quantitative imaging of H_2_S dynamics in complex biological environments [78].

### 2.4. Other Auxiliary Sensing Mechanisms

Aggregation-induced emission (AIE) is a photophysical phenomenon in which a fluorophore shows weak or negligible fluorescence in solution but enhanced fluorescence upon aggregation [79]. First reported by Tang et al. in 2015, AIE has since been widely investigated for fluorescence sensing [80]. The enhanced fluorescence in the aggregated state is generally attributed to the restriction of intramolecular motions (RIM), which reduces nonradiative decay and promotes radiative emission [81]. AIE-based fluorescent probes have also been developed using the 1,8-naphthalimide scaffold for H_2_S detection [81]. Compared with conventional fluorescent probes, AIE-based systems can provide high photostability and low background fluorescence, making AIE a useful strategy for improving the sensitivity of fluorescent probes [82].

Excited-state proton transfer (ESPT), including excited-state intramolecular proton transfer (ESIPT), is another photophysical mechanism used in fluorescence sensing. In this process, photoexcitation promotes proton transfer from a proton donor to a nearby proton acceptor, generating an excited-state species with different electronic and fluorescence properties [83]. ESPT/ESIPT can produce a large Stokes shift, which reduces self-absorption and interference from excitation light, making this mechanism attractive for fluorescence sensing [84]. This mechanism has also been incorporated into fluorescent probes for H_2_S detection, in which an H_2_S-mediated chemical transformation of the recognition group alters the proton-transfer pathway and produces a fluorescence response [85]. The large Stokes shift is particularly beneficial for ESPT/ESIPT-based probes in biological environments because it can reduce background interference and improve fluorescence imaging [86].

Overall, although AIE and ESPT/ESIPT provide alternative strategies for modulating fluorescence in H_2_S probes, they have been less frequently used as independent sensing mechanisms. In many reported systems, AIE or ESPT/ESIPT is combined with established mechanisms such as PET or ICT, where it serves as an additional process to modulate the fluorescence response rather than as the primary sensing mechanism. This combination provides additional opportunities to improve the fluorescence response and sensing performance of H_2_S probes.

## 3. Reaction-Based Recognition for H_2_S

### 3.1. Detecting H_2_S by H_2_S-Triggered Reduction Reactions

**Figure 2:**
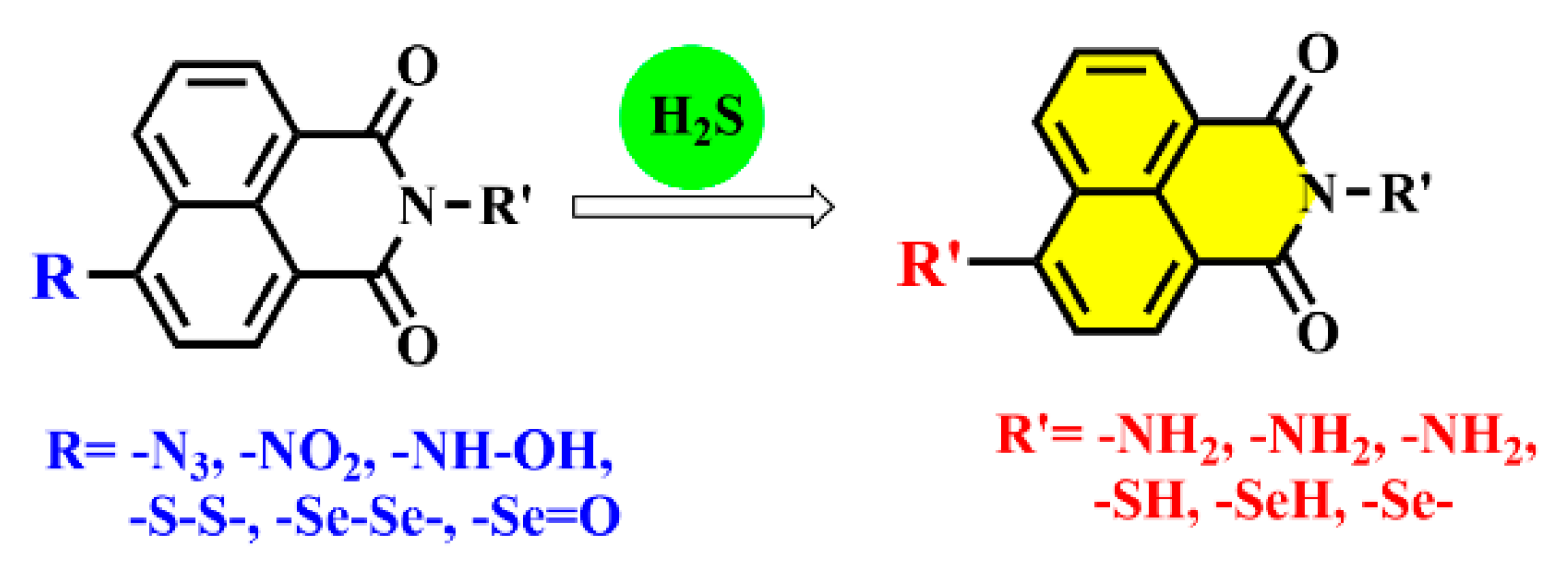
Reduction of azides, nitro groups, hydroxylamines, disulfides, diselenides, or selenoxides groups appended to the 1,8-naphthalimide scaffold as a strategy for the design of fluorescent probes for H_2_S detection.

The intrinsic reducing capability of H_2_S offers a robust and rational strategy for fluorescent probe design through the incorporation of reduction-responsive moieties, such as aryl azides, nitro groups, hydroxylamines, disulfides, diselenides, or selenoxides into the 1,8-naphthalimide scaffold. Upon selective reduction by H_2_S, these groups are converted into electron-donating groups that modulate photophysical pathways, including ICT, PET, or FRET. Such reduction-induced changes generate measurable fluorescence turn-on or ratiometric responses, enabling quantitative detection of H_2_S (Figure 2). This reaction-based mechanism provides high sensitivity and selectivity under physiological conditions, facilitating monitoring of H_2_S dynamics in complex biological environments.

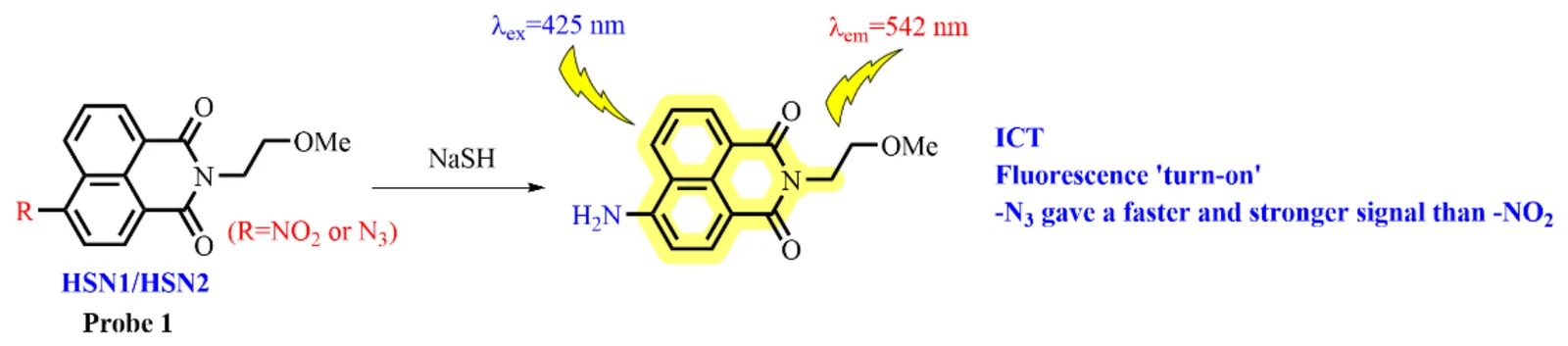



In 2012, Montoya et al. reported probe **1** as a 1,8-naphthalimide-based fluorescent probe for H_2_S detection [87]. In this design, either a nitro or an azide group was introduced at the C4 position of the 1,8-naphthalimide core as the recognition unit. Upon exposure to H_2_S, both functional groups undergo reduction to the corresponding amine, thereby enhancing the electron-donating character at C4 that produces a fluorescence “turn-on” response. Both probes exhibited pronounced fluorescence enhancement in the presence of H_2_S and demonstrated high selectivity over other biologically relevant thiols, including cysteine and glutathione, even at millimolar concentrations (up to 10 mM). Notably, the azide-containing probe displayed faster reaction kinetics, higher conversion efficiency, and higher selectivity than its nitro analogue. Furthermore, both probes were successfully applied in HeLa cells, generating strong intracellular fluorescence signals and confirming their biocompatibility. This study demonstrated an efficient sensing method based on a 1,8-naphthalimide scaffold for detecting H_2_S. This conceptual strategy established a foundational platform that subsequently inspired numerous 1,8-naphthalimide-based H_2_S probes employing similar reduction-triggered mechanisms.

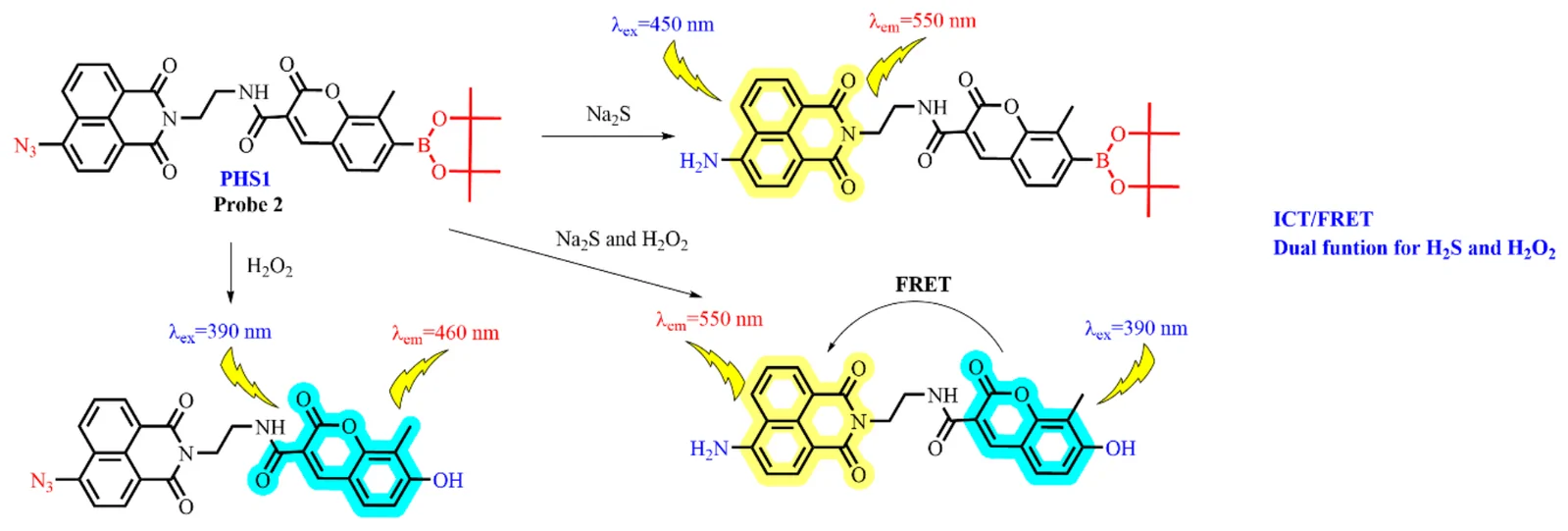



Velusamy et al. reported probe **2**, which employs an azide reduction strategy for the selective detection of H_2_S. In the presence of Na_2_S, the azide group at the C4 position of the 1,8-naphthalimide core is reduced to an amine, resulting in fluorescence enhancement at 550 nm through an intramolecular charge transfer (ICT) process in HEPES buffer (1% DMSO, pH 7.4). In addition, a coumarin unit bearing a boronate ester was introduced at the *N*-imide position. Upon exposure to H_2_O_2_, the boronate ester is converted to a phenolic hydroxyl group, leading to fluorescence enhancement at 460 nm. When probe **2** is treated with H_2_S and H_2_O_2_, a FRET process occurs between the coumarin donor and the 1,8-naphthalimide acceptor. Probe **2** exhibits high stability and selectivity in the presence of various analytes and across a broad pH range (4–9.5). Furthermore, cell viability assays indicate that the probe shows low cytotoxicity toward colon cancer cells (HT29), supporting its suitability for cellular imaging applications [88].

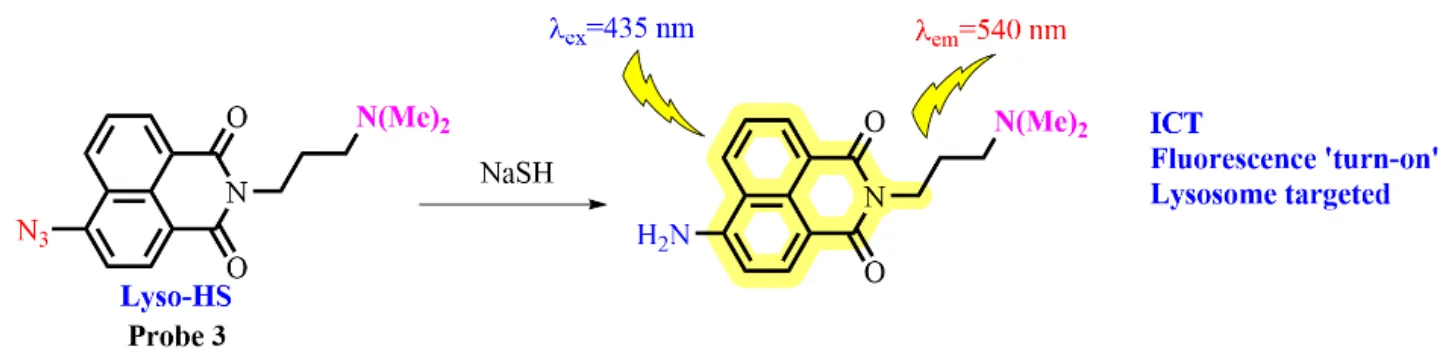



Due to its diverse biological roles, H_2_S is distributed across multiple subcellular organelles at varying concentrations. Wu et al. reported probe **3** as a lysosome-targeted fluorescent probe for H_2_S detection. In this design, an *N,N*-dimethylpropyl imide moiety was introduced as the lysosomal targeting unit, facilitating selective accumulation within lysosomes. Upon treatment with NaHS in PBS buffer (20 mM, 1% DMSO, pH 7.4), probe **3** exhibited fluorescence emission centered at 540 nm, with an approximately 15-fold enhancement in intensity within 60 min. Notably, incorporation of the *N,N*-dimethylpropyl group significantly improved the selectivity of probe **3** toward H_2_S compared to the corresponding *n*-butyl-substituted analogue. Under physiological conditions, probe **3** carries a positive charge, which enhances its aqueous solubility and promotes a faster and more sensitive fluorescence response to H_2_S [89].

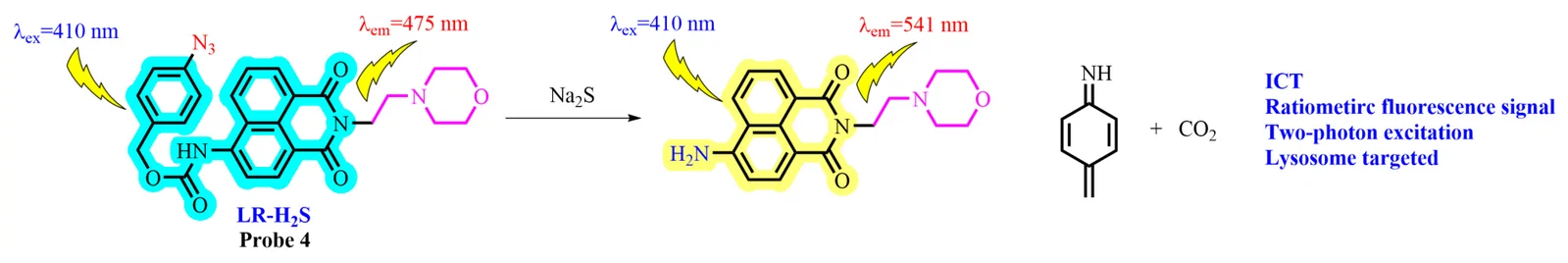



Under two-photon excitation at 840 nm, probe **4** exhibited an approximately 80-fold increase in the fluorescence intensity ratio (F_541_/F_475_), demonstrating a broad linear response range from 25 to 2500 μM. The detection limit was determined to be 0.70 μM. Probe **4** displayed excellent selectivity toward H_2_S over other biologically relevant interfering species and showed low cytotoxicity under the tested conditions. Cell imaging studies confirmed that probe **4** can detect both endogenous and exogenous H_2_S in the lysosomes of living SGC-7901 cells [90].

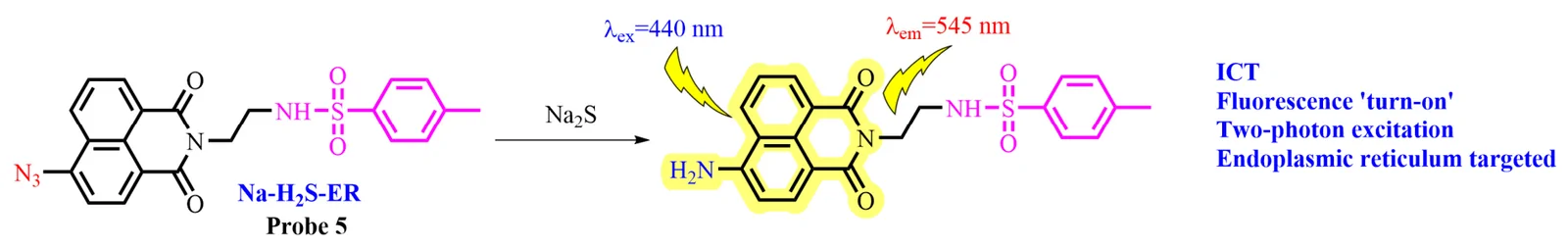



Tang et al. reported probe **5** as an endoplasmic reticulum (ER)-targeted fluorescent probe for H_2_S detection. In the presence of Na_2_S, probe **5** exhibited fluorescence emission at 545 nm, attributed to activation of an intramolecular charge transfer (ICT) process. The probe showed selective response toward Na_2_S, low cytotoxicity, and stable performance at physiological pH conditions. Probe **5** was applied to monitor H_2_S levels in the ER of living cells. It was further utilized in tissue imaging and zebrafish imaging experiments [91].

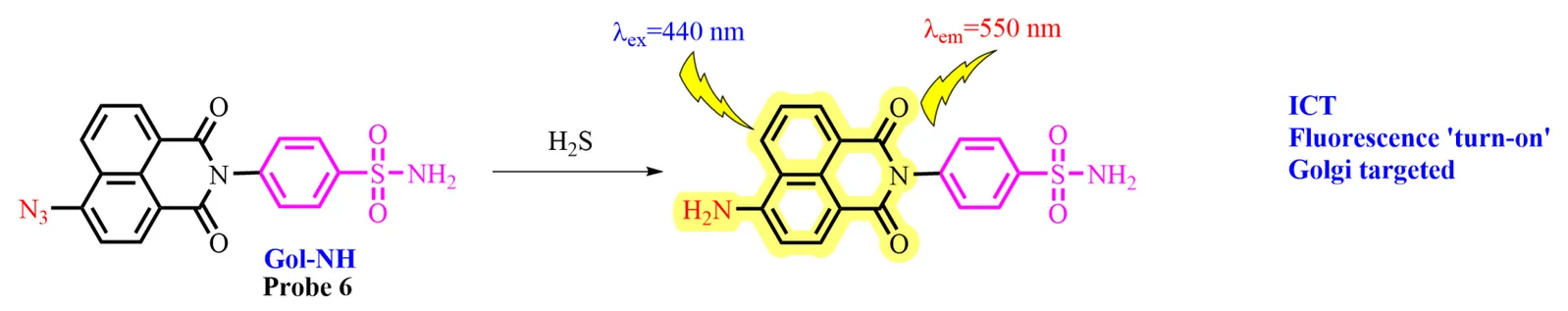



In 2020, Zhu et al. reported probe **6**, a Golgi-targeted fluorescent probe for H_2_S, designed to investigate Golgi stress. Their study demonstrated that H_2_S can serve as a biomarker for monitoring the Golgi stress response. Probe **6** was employed to detect both endogenous and exogenous H_2_S in zebrafish models. To confirm the Golgi-specific localization of probe **6**, fluorescence colocalization experiments were performed using a commercial Golgi-Tracker Red dye. The imaging results showed strong overlap between the two signals, with an average Pearson’s colocalization coefficient of 0.92, indicating efficient targeting to the Golgi apparatus [92].

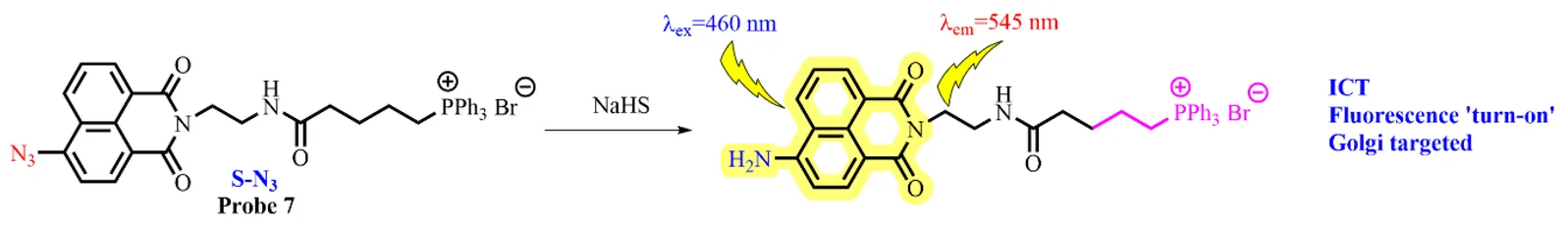



Shi et al. reported probe **7** as a mitochondria-targeted fluorescent probe for detecting H_2_S. Probe **7** exhibited high sensitivity toward H_2_S, with a detection limit of 1 μM at a signal-to-noise ratio (S/N) of 3:1, and a limit of quantification (LOQ) of 4.2 μM at an S/N of 10:1. The probe maintained stable sensing performance across a broad pH range (3–11), in various buffer systems, and demonstrated excellent photostability. Moreover, probe **7** displayed low cytotoxicity in MCF-7 cells, supporting its suitability for cellular imaging applications [93].

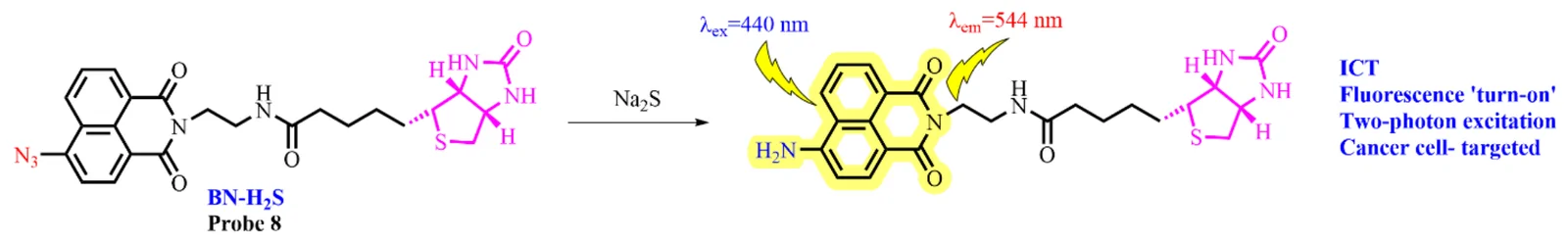



Song et al. developed probe **8** as a cancer cell–targeted two-photon fluorescent probe for H_2_S detection. In the molecular design, biotin was incorporated as a targeting moiety to facilitate selective uptake by cancer cells. Upon reaction with H_2_S, probe **8** exhibited a fluorescence “turn-on” response with an emission maximum at 544 nm. The probe demonstrated high sensitivity, with a detection limit of 71 nM, and excellent selectivity toward H_2_S over other biologically relevant species. Probe **8** was successfully applied to two-photon fluorescence imaging of H_2_S in living cancer cells, while displaying a comparatively weak response in normal cells [94].

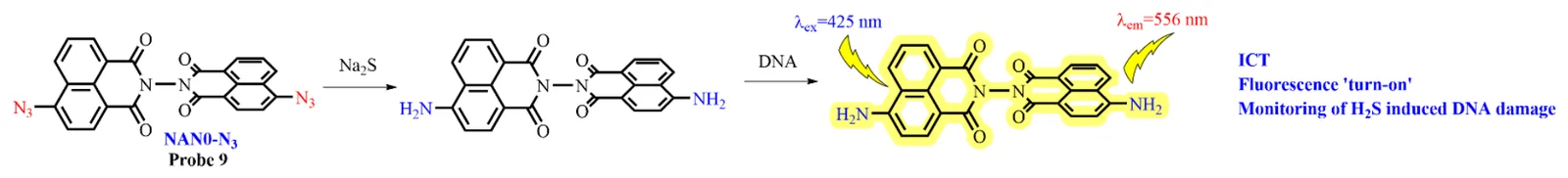



A dual-locked fluorescent probe **9** was developed for the selective monitoring of H_2_S-induced DNA damage in living cells by Wang et al. Spectroscopic analyses combined with theoretical calculations demonstrated that probe **9** binds strongly to DNA via minor-groove interactions, inducing conformational restriction that suppresses nonradiative decay pathways. Concurrently, H_2_S triggers a structural transformation of the probe, further modulating its photophysical properties. In this system, H_2_S (≥1.9 × 10^−2^ mM) and DNA (≥2.2 × 10^−2^ μg mL^−1^) function as dual keys, synergistically activating a highly sensitive fluorescence response through cooperative structural modulation. Probe **9** enables the selective visualization of H_2_S-induced damage in both nDNA and mtDNA [95].

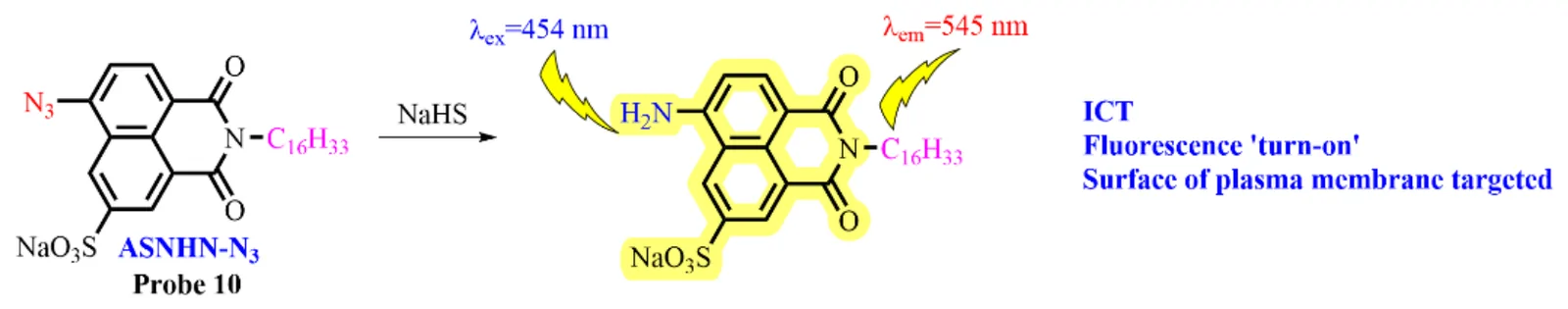



Probe **10** was designed as a cell surface-targeted H_2_S probe to investigate extracellular H_2_S release by Fu et al. In a pH 7.4 buffer at 37 °C, probe **10** exhibited weak basal fluorescence but underwent a marked fluorescence enhancement upon reaction with H_2_S. A good linear correlation between fluorescence intensity and NaHS concentration was observed over the range of 0–10 μM, with a detection limit of 0.75 μM. Through the incorporation of a lipid anchor and a hydrophilic sulfonate group into the 1,8-naphthalimide scaffold, probe **10** was effectively localized to the plasma membrane of living cells. This membrane-anchoring design enabled real-time monitoring of H_2_S efflux as it diffuses across the cell membrane. Moreover, the probe was successfully applied to deep-tissue imaging using two-photon microscopy [96].

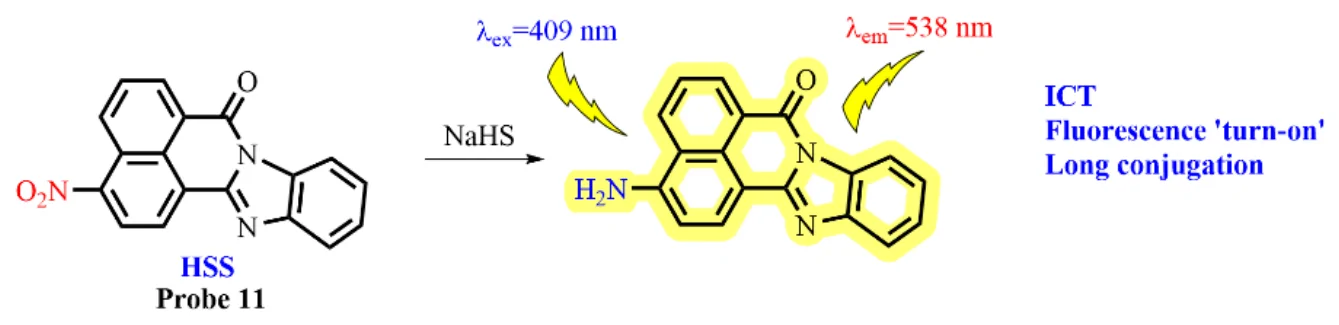



In 2015, Bamesberger et al. reported probe **11**, which was synthesized in a single step by reducing a nitro group to the corresponding amine. Probe **11** exhibited high sensitivity and selectivity toward H_2_S over other potential reducing agents. Upon exposure to H_2_S, a 30-fold fluorescence enhancement was observed, producing a “turn-on” response base on ICT mechanism and showed a linear correlation with H_2_S concentration in the range of 0–100 μM. Although nitro reduction is generally less efficient than azide reduction, probe **11** demonstrated a relatively rapid response, with the reduction process completed within 15 min in aqueous media [97].

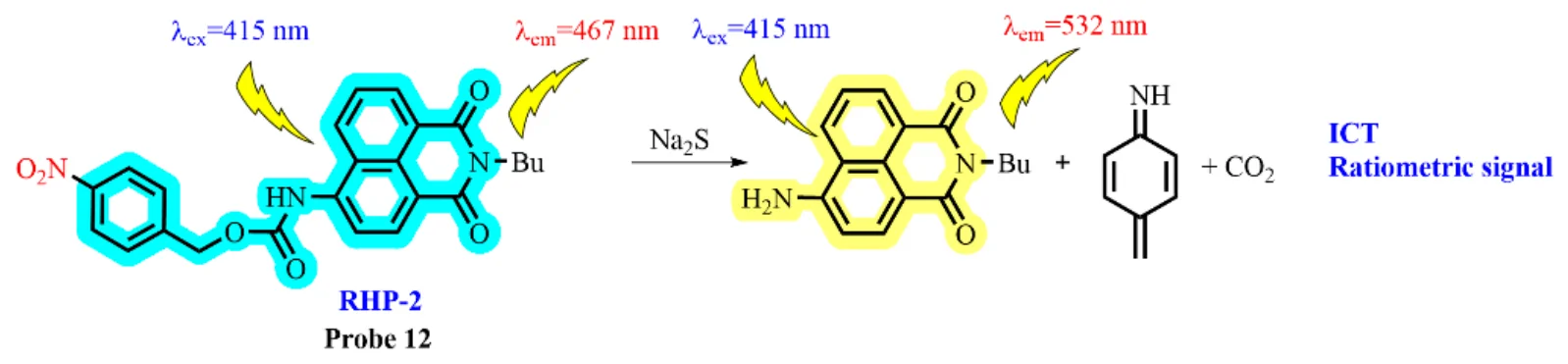



Zhang et al. reported a ratiometric probe **12** based on nitro-group reduction. Upon reduction of the nitro moiety to the corresponding amine, a subsequent self-immolative cleavage was triggered, yielding 4-amino-1,8-naphthalimide. The released fluorophore exhibited fluorescence emission at 532 nm (*Φ* = 0.13), arising from an enhanced ICT process. This reduction–cleavage sequence has been widely employed as an effective sensing strategy for H_2_S detection. Probe **12** was successfully applied to the detection and imaging of endogenous H_2_S in MCF-7 cells and for the quantification of endogenous H_2_S levels in the mouse hippocampus [98].

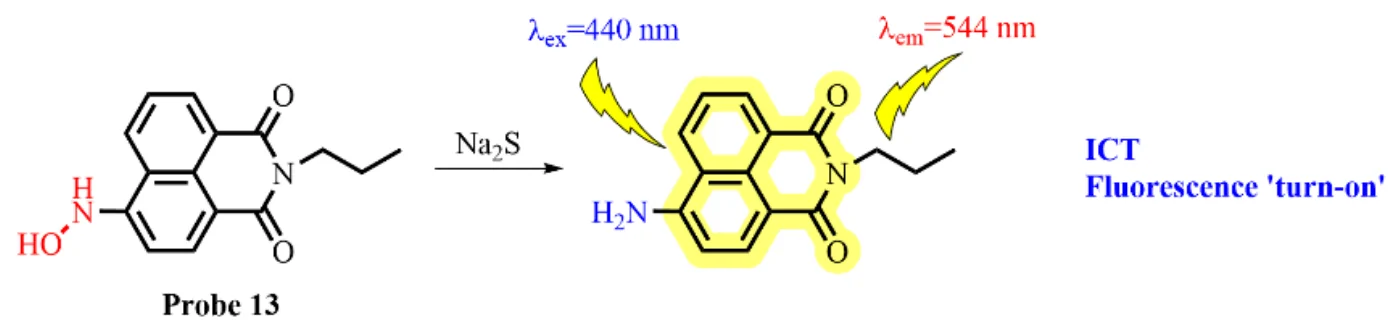



Hydroxylamine has also been employed as a recognition unit for H_2_S detection, operating through an H_2_S-triggered reduction mechanism analogous to those of azide- and nitro-based probes. Based on this strategy, Xuan et al. developed probe **13**, which exhibited high selectivity and sensitivity toward H_2_S at submicromolar concentrations. Upon incubation of 2.0 μM probe **13** with 10.0 μM H_2_S (Na_2_S as the donor), fluorescence emission was monitored over 180 min. A pronounced fluorescence enhancement at 544 nm was observed, corresponding to more than a 13-fold increase in intensity. The response rate was accelerated under mildly basic conditions; however, compared with other reported H_2_S probes, probe **13** displayed relatively slow reaction kinetics. Probe **13** was successfully applied for H_2_S detection in astrocytes and showed no obvious cytotoxicity [99].

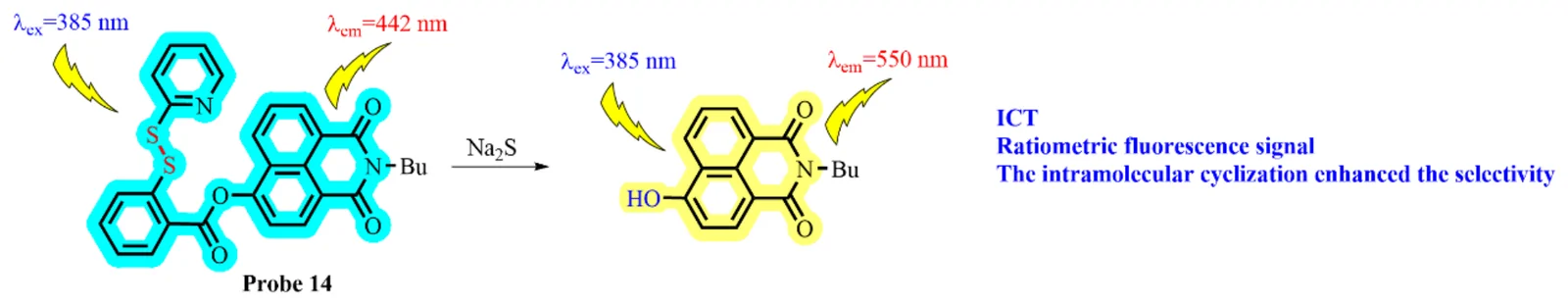



Zhang et al. reported probe **14**, which operates via an H_2_S-triggered reduction of a disulfide bond followed by an intramolecular cyclization reaction. In DMF/water (1:9, *v*/*v*; PBS buffer, pH 7.4), probe **14** exhibited a 30-fold enhancement in the emission ratio (F_550_/F_442_) toward H_2_S within 3 min of incubation. The detection limit for Na_2_S was determined to be 0.87 μM. Upon treatment of probe **14** (10 μM) with Na_2_S (50 μM) in DMF/water (1:9, *v*/*v*; 50 mM PBS, pH 7.4) containing 1 mM CTAB, maximal fluorescence enhancement was achieved within 3 min, indicating a rapid response. Cellular imaging studies demonstrated that probe **14** possesses good membrane permeability and can detect intracellular H_2_S. The reduction–cyclization mechanism confers high selectivity toward H_2_S over other biothiols [100].

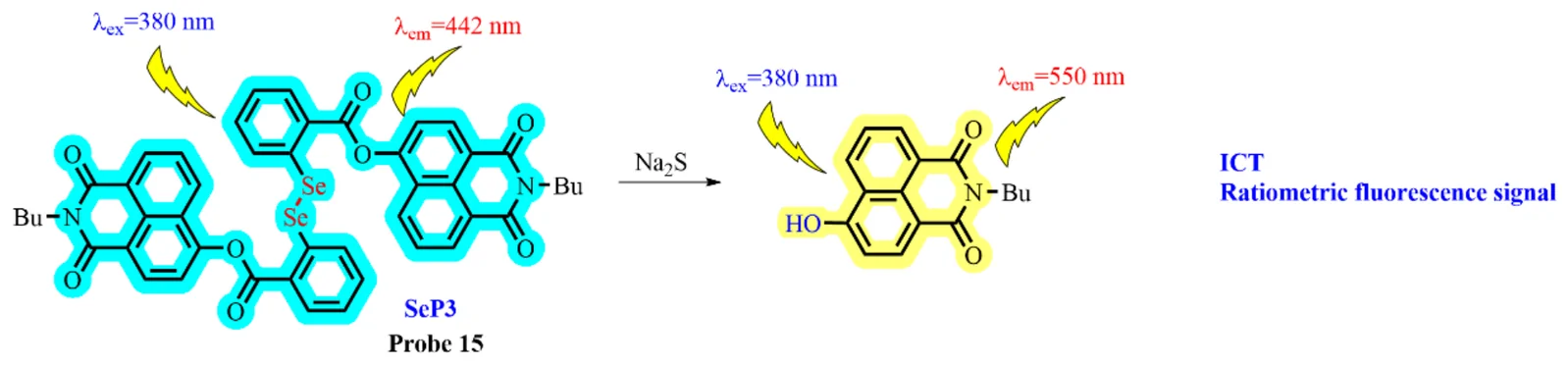



Cleavage of diselenide bonds triggered by H_2_S has also been widely exploited for selective detection of H_2_S. Zhang et al. reported a ratiometric fluorescent probe **15** based on H_2_S-mediated reduction of a diselenide moiety, followed by intramolecular cyclization. Probe **15** exhibited high sensitivity and selectivity toward H_2_S over other reactive sulfur species and was successfully applied to detect H_2_S in bovine plasma as well as for bioimaging in live HeLa cells. Notably, this strategy significantly minimized false-positive signals arising from endogenous biothiols [101].

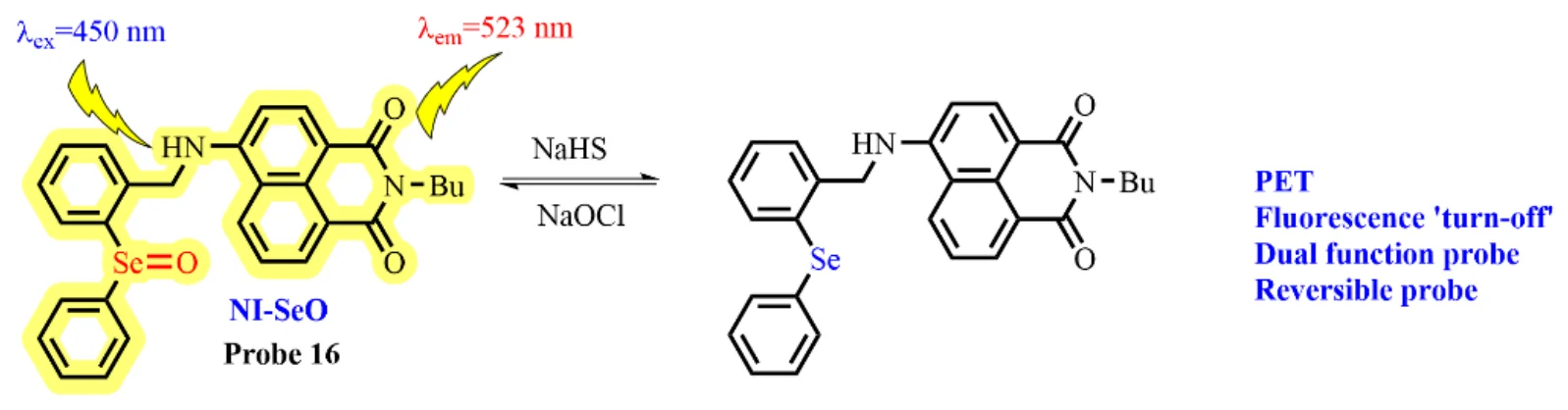



Lou et al. developed a selenoxide-based fluorescent probe **16** that exploits reversible selenide/selenoxide redox interconversion. The design enables bidirectional responsiveness toward both hypochlorous acid (HOCl) and hydrogen sulfide. Oxidation of the selenide to the corresponding selenoxide by HOCl induces a fluorescence turn-on response, whereas subsequent reduction by H_2_S restores the selenide form, resulting in fluorescence quenching. This reversible redox cycle allows dynamic monitoring of oxidative and reductive processes in biological systems. Probe **16** was successfully applied to visualize HOCl generation and antioxidant-mediated redox regulation in living RAW264.7 cells and in mouse models, demonstrating its utility for in situ redox imaging [102].

### 3.2. Detecting H_2_S by Nucleophilic Aromatic Substitution (S_N_Ar) Reactions

**Figure 3:**
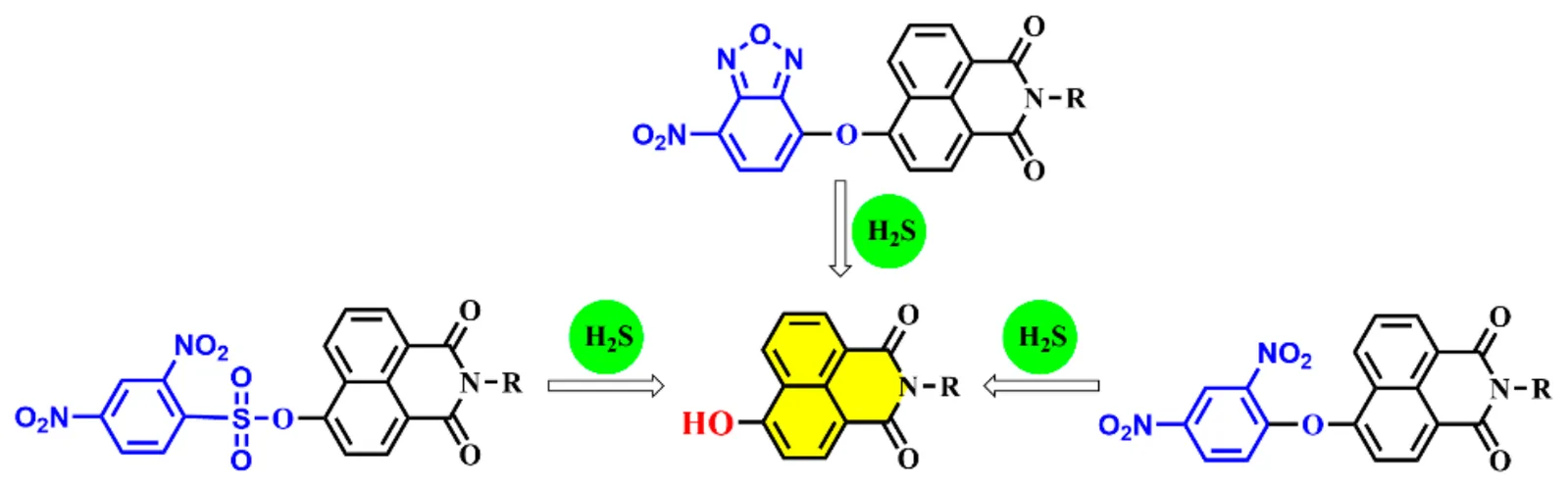
Nucleophilic aromatic substitution (S_N_Ar) reactions are used as the recognition mechanism for detecting H_2_S.

In aqueous media, H_2_S exists predominantly as the hydrosulfide anion (HS^−^), which can account for up to ~80% of the total sulfide species under physiological conditions [103]. HS^−^ is a relatively strong nucleophile, a property that can be exploited to differentiate it from other biological thiols such as Cys and GSH, which generally display lower nucleophilicity under comparable conditions. H_2_S-triggered nucleophilic aromatic substitution (S_N_Ar) reactions have therefore been widely employed as the recognition mechanism in reaction-based H_2_S fluorescent probes. In these systems, electron-deficient aromatic groups are incorporated to facilitate nucleophilic attack by HS^−^, enabling efficient S_N_Ar substitution. Commonly used scaffolds include 2,4-dinitrobenzenesulfonate (DNBS), 2,4-dinitrobenzoate (DNBE), and 7-nitrobenzofurazan ester (NBDE), which are frequently incorporated into probe designs for S_N_Ar-based H_2_S detection [104]. Due to its strong nucleophilicity, HS^−^ selectively reacts with these electrophilic motifs, resulting in cleavage and generation of a “turn-on” fluorescence signal (Figure 3).

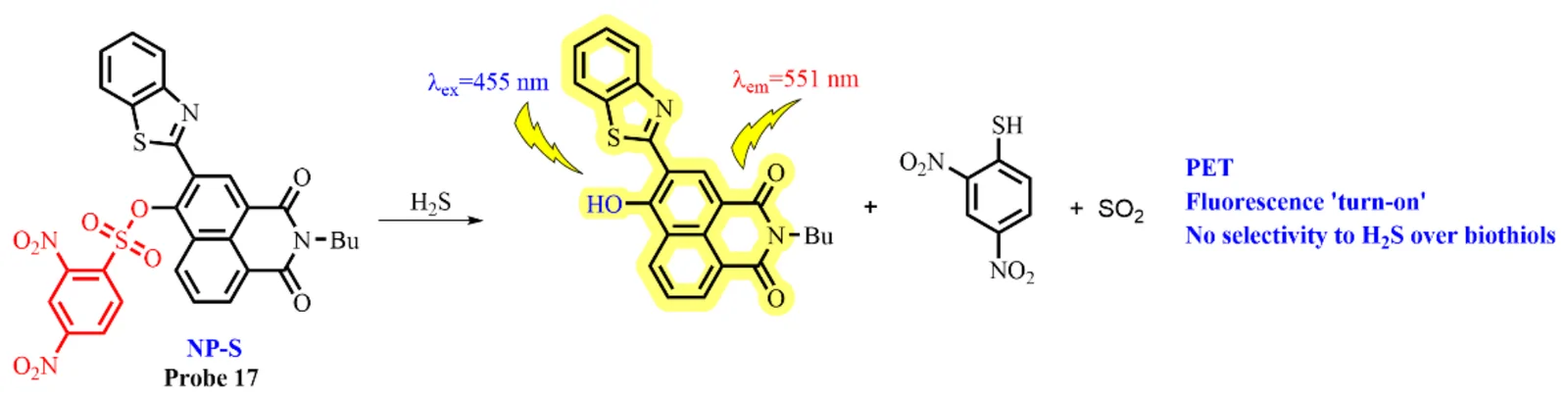



In 2019, Xu et al. reported probe **17**, a DNBS-based fluorescent probe for detecting H_2_S. The probe features a benzothiazole substituent at the C3 position of the 1,8-naphthalimide core and a DNBS group at C4, which serves as both the recognition unit and fluorescence quencher through a photoinduced electron transfer (PET) mechanism. Upon exposure to H_2_S or biothiols, the DNBS group undergoes a nucleophilic aromatic substitution (S_N_Ar) reaction, leading to cleavage of the sulfonyl group and restoration of fluorescence. As a result, probe **17** exhibits a rapid fluorescence enhancement with emission at 551 nm in aqueous solution (25 mM PBS, pH 7.4, containing 20% EtOH). Although the benzothiazole unit extends the π-conjugation, it does not produce a significant red shift in the emission wavelength. Notably, probe **17** responds to H_2_S, Cys, GSH, and Hcy without clear selectivity toward H_2_S. This observation is noteworthy because some DNBS-based probes display high selectivity for H_2_S over biothiols despite only minor structural differences, highlighting the strong sensitivity of the 1,8-naphthalimide fluorophore to structural modifications. In addition, solvent composition, particularly the type of organic co-solvent, can significantly influence sensing performance. Confocal fluorescence imaging further demonstrated the applicability of probe **17** for detecting thiols and H_2_S in HeLa cells and zebrafish [105].

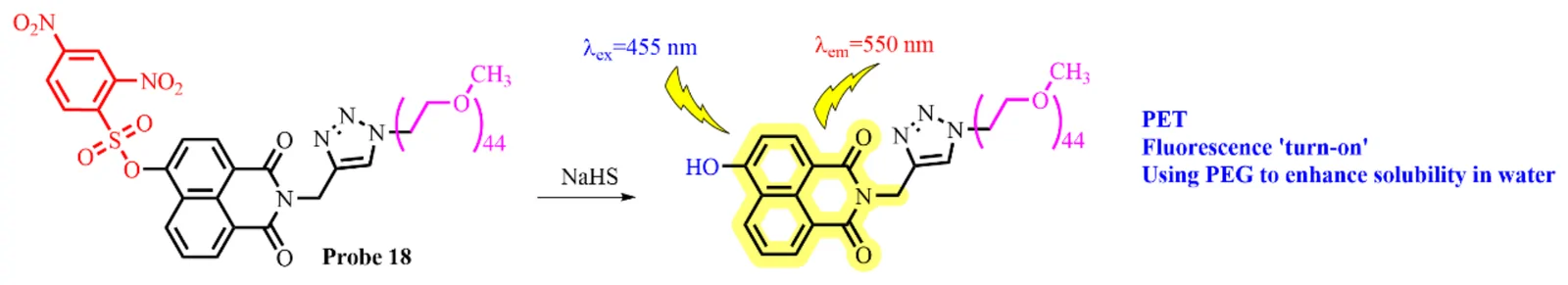



Xiao et al. reported probe **18**, which incorporates a long hydrophilic polyethylene glycol (PEG) chain to enhance water solubility. Owing to this structural modification, probe **18** can effectively detect H_2_S in pure aqueous media and in H_2_S gas in air. The probe exhibits high sensitivity toward H_2_S, with a limit of detection (LOD) as low as 9.95 × 10^−9^ M. In addition, probe **18** was applied as an indicator for monitoring food spoilage by detecting H_2_S produced during microbial growth. For practical and portable sensing applications, the probe was further fabricated into test paper strips using an immersion–coating method. In the presence of H_2_S, the strips display a distinct yellow color change, enabling simple visual detection without the need for sophisticated instrumentation [106].

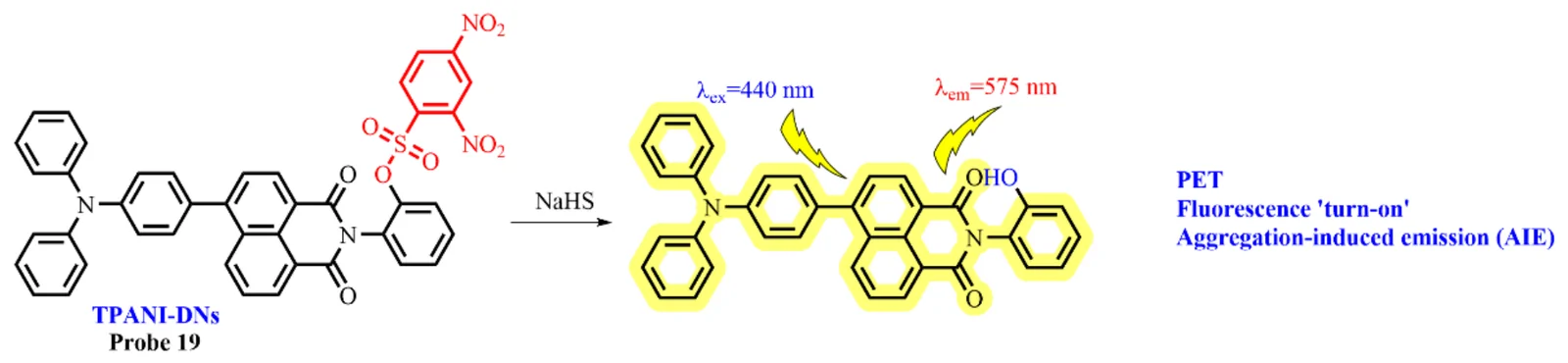



Probe **19** is an aggregation-induced emission (AIE)-based probe for H_2_S developed by Liu et al. In its native state, probe 19 is nonfluorescent due to donor-excited photoinduced electron transfer (d-PET). Upon reaction with H_2_S, cleavage of the recognition unit generates twisted D–π–A products, which subsequently form aggregates exhibiting pronounced AIE behavior. As a result, a significant turn-on fluorescence signal is observed. Probe 19 displays high sensitivity and selectivity toward H_2_S, along with a rapid response time. The sensing mechanism and photophysical properties were further investigated using time-dependent density functional theory (TDDFT) calculations. In addition, probe 19 was fabricated into paper strips for on-site detection of H_2_S in practical applications. The strongly electron-withdrawing 2,4-dinitrobenzenesulfonyl (DNBS) group is commonly introduced onto the naphthalene ring of 1,8-naphthalimide as a fluorescence-quenching recognition unit. In this system, probe 19 demonstrates that the DNBS group can also effectively quench fluorescence when attached to the *N*-imide position of the fluorophore [107].

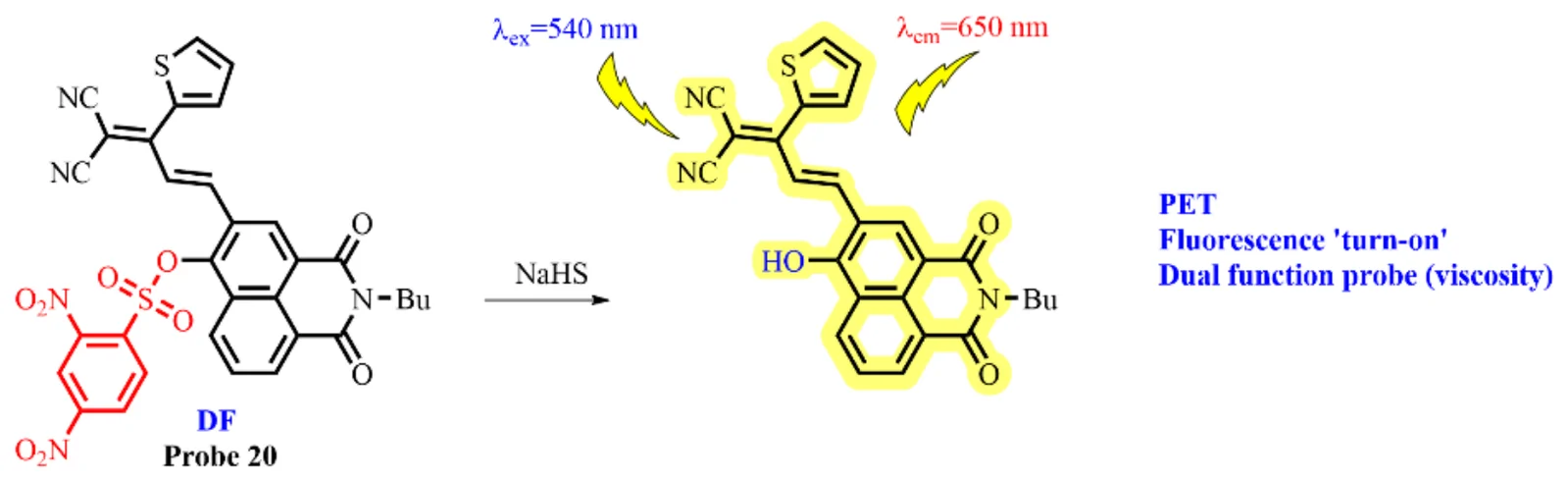



Shen et al. reported probe **20**, a fluorescent probe with dual functionality for detecting H_2_S and medium viscosity. In DMSO/PBS buffer (*v*/*v* = 1:99, pH 7.4), probe **20** exhibits near-infrared fluorescence emission at 650 nm in the presence of H_2_S. A good linear relationship between fluorescence intensity and H_2_S concentration was observed in the range of 0–10 μM, with a detection limit of 79 nM. The presence of H_2_S induces an 18–20-fold fluorescence enhancement, while other biothiols, including Cys, GSH, and Hcy, produce negligible fluorescence changes at 650 nm. In addition to H_2_S sensing, probe **20** responds to variations in viscosity. When the viscosity of the medium increased by adding glycerol (from 1.03 CP to 954.52 CP), the fluorescence intensity increased by approximately 50-fold at 580 nm. Probe **20** was further applied to fluorescence imaging of viscosity and H_2_S in HeLa cells and zebrafish [108].

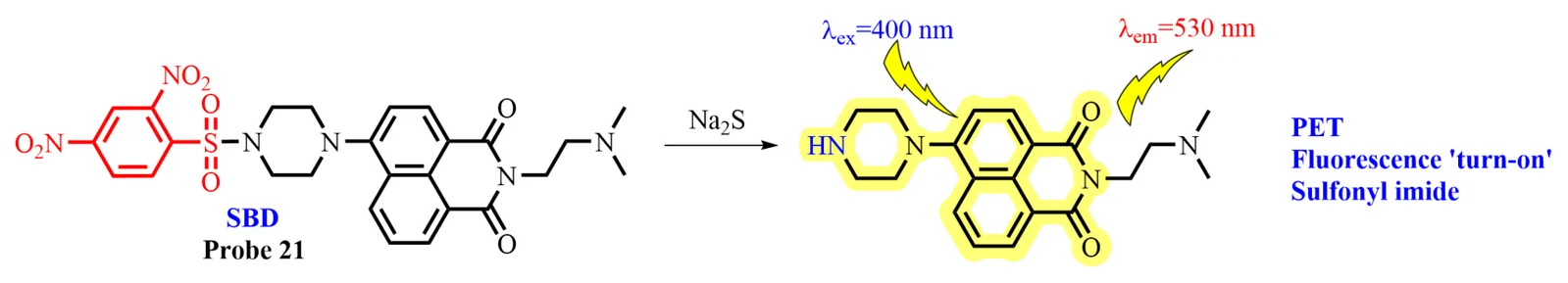



In general, imides exhibit lower reactivity than esters. However, dinitrobenzenesulfonyl (DNBS) imide derivatives can still react with NaHS and function as recognition units for H_2_S detection. Shi et al. reported probe **21**, which employs a DNBS imide moiety for H_2_S sensing. Upon reacting with H_2_S, probe **21** induces a distinct color change from yellow to pink, enabling naked-eye detection. In addition, the probe displays a significant fluorescence enhancement at 530 nm (approximately 45-fold). Probe **21** also shows good selectivity and strong anti-interference capability toward H_2_S. The limit of detection for H_2_S was 152 nM. Furthermore, the probe exhibits low cytotoxicity and has been successfully applied for fluorescence imaging of exogenous H_2_S in CaKi-1 cells [109].

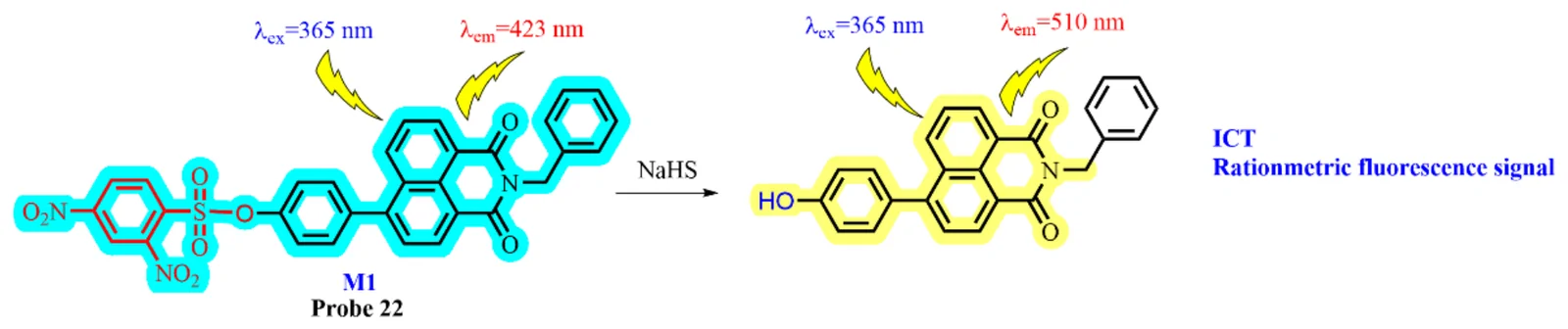



Dvorak et al. reported probe **22**, in which the DNBS group is not directly attached to the naphthalene moiety; instead, a phenyl spacer is inserted between the DNBS unit and the naphthalene ring. Probe **22** exhibits a ratiometric fluorescence response toward H_2_S. In the absence of H_2_S, the probe shows an emission peak at 423 nm, which shifts to 510 nm after exposure to H_2_S in aqueous solution (MeCN/H_2_O = 1:1). Typically, the DNBS group acts as a strong quencher of 1,8-naphthalimide fluorescence. However, the presence of the phenyl spacer reduces the quenching efficiency of the DNBS group. Upon addition of H_2_S, cleavage of the DNBS moiety removes the quenching effect, producing a new emission band at 510 nm. Probe **22** exhibits a limit of detection (LOD) of 0.64 μM and responds rapidly to H_2_S, with the fluorescence intensity at 510 nm reaching its maximum within 10 min [110].

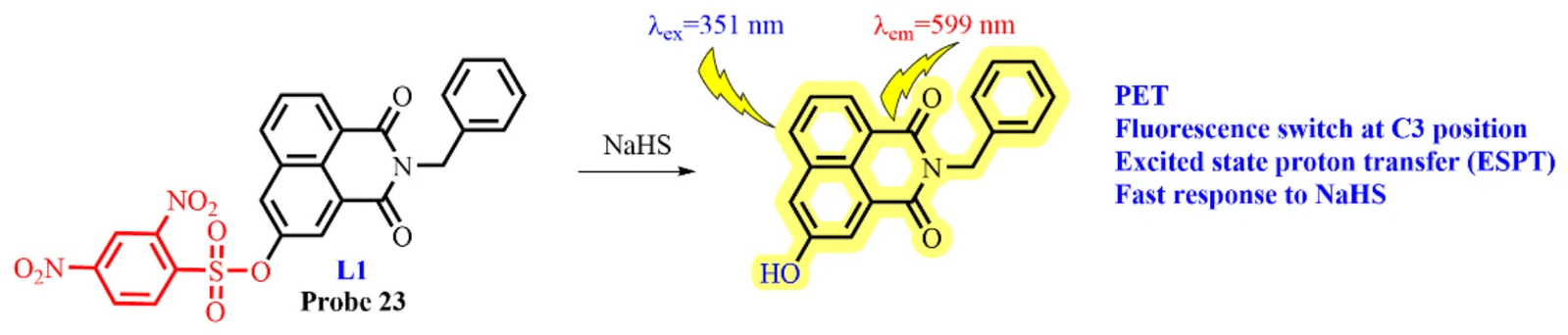



Most of 1,8-naphthalimide-based H_2_S probes included in this review employ the C4 position of the naphthalene ring to introduce a fluorescent switch or recognition moiety, thereby generating a fluorescence response. To the best of our current knowledge, probe **23** is the first example in which the C3 position of the naphthalimide scaffold was utilized for probe design. Owing to the excited-state proton transfer (ESPT) characteristics of 3-hydroxy-1,8-naphthalimide, probe **23** (10 μM) exhibits a rapid response to NaHS (80 μM), with fluorescence enhancement occurring almost instantaneously in DMSO-containing media (40–90%) at pH 7.4. Compared with conventional C4-substituted probe s, which typically emit around 550 nm, the C3-substituted probe displays a red-shifted emission at 599 nm in DMSO/phosphate buffer (0.01 M, pH 7.4, 3:7 *v*/*v*). In addition, the DNBS-based probe responds significantly faster to NaHS than the corresponding DNBE-based probe under identical conditions [85].

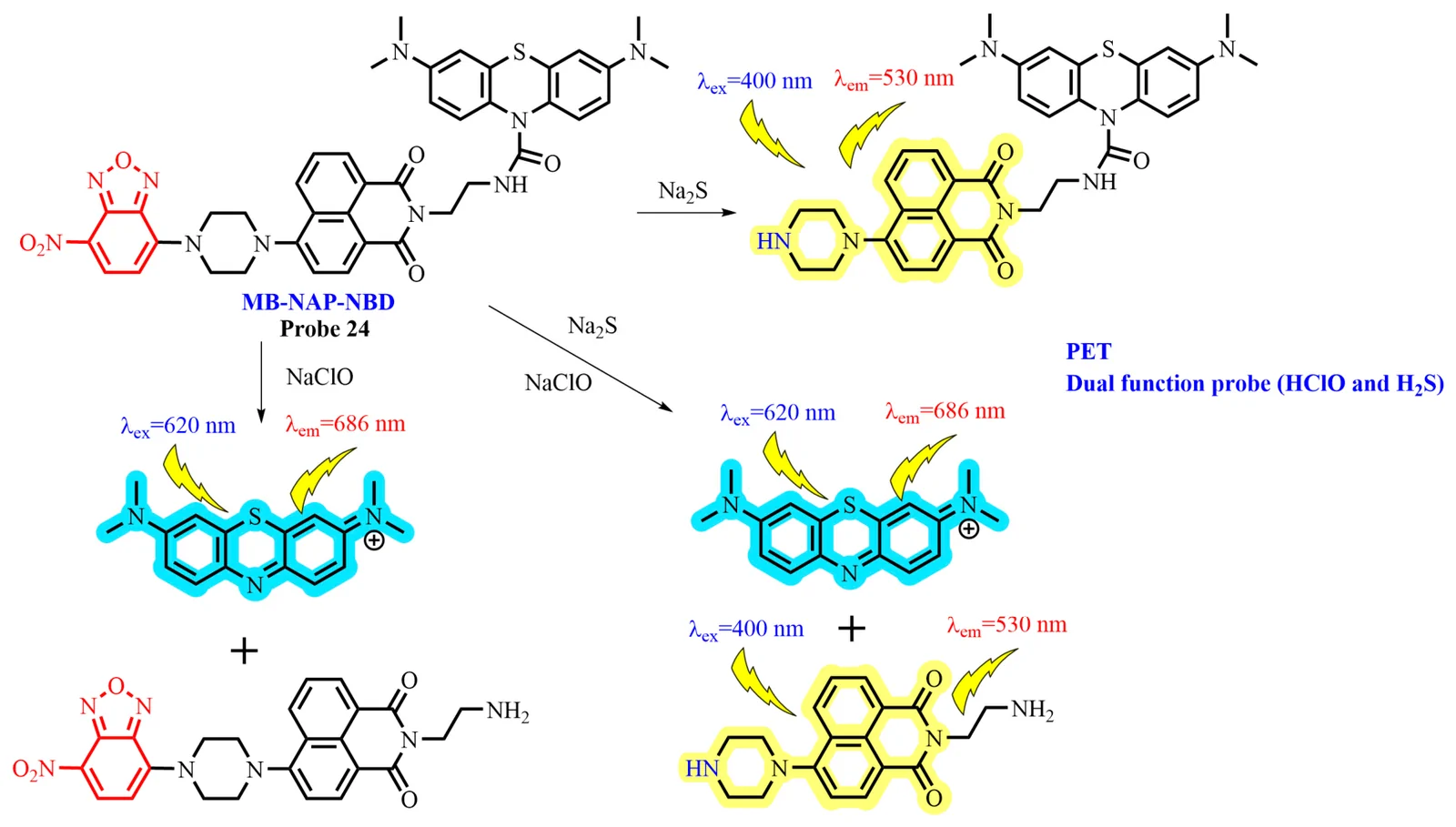



Cheng et al. reported probe **24**, a fluorescent probe based on the NBD scaffold. In the presence of H_2_S, probe **24** exhibits a turn-on fluorescence response at 530 nm in PBS buffer (pH 7.4). The fluorescence intensity reaches its maximum after 40 min incubation with Na_2_S (50 μM) in PBS buffer (pH 7.4) containing 20% DMSO as a cosolvent. Probe **24** has been applied for the detection of exogenous H_2_S in HeLa cells and zebrafish. In addition to H_2_S sensing, probe **24** functions as a dual-response probe, as it can also detect hypochlorous acid (HOCl), producing a “turn-on” fluorescence signal at 664 nm [111].

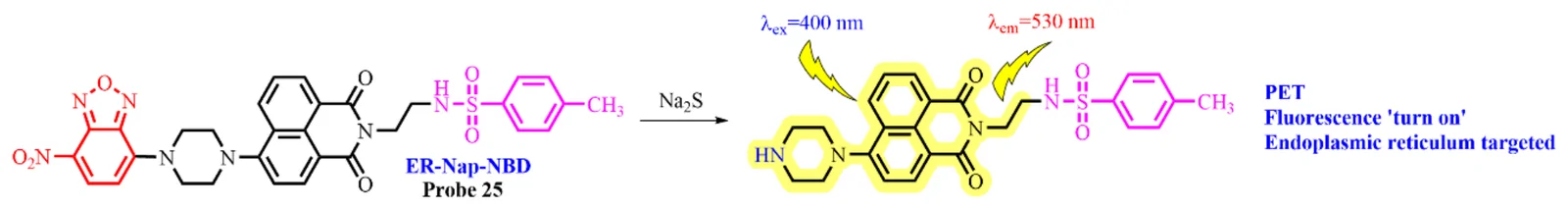



Probe **25**, reported by Kafuti et al., is an endoplasmic reticulum (ER)-targeted fluorescent probe based on an NBD scaffold, exhibiting a turn-on fluorescence emission at 530 nm. The probe shows a limit of detection (LOD) of 5.2 μM and a strong linear correlation (R^2^ = 0.99797) between the fluorescence intensity at 530 nm and Na_2_S concentration in the range of 0–450 μM in EtOH/PBS (1:1, *v*/*v*, pH 7.4) solution. Probe **25** displays a fluorescence enhancement upon incubation with Na_2_S over a pH range of 4–8. Co-localization fluorescence imaging in MCF-7 cells confirmed the endoplasmic reticulum-targeting capability of probe **25**. In addition, probe **25** was used to perform fluorescence imaging of H_2_S in zebrafish [112].

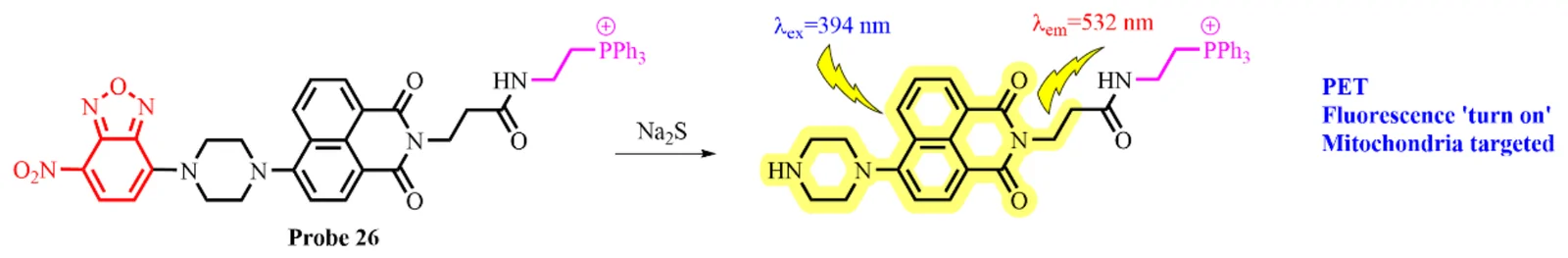



Pak et al. reported probe **26**, a turn-on mitochondria-targeted fluorescent probe for hydrogen sulfide. Probe **26** is based on the selective thiolysis of an NBD unit appended to a piperazine-linked 1,8-naphthalimide scaffold. In the presence of Na_2_S, probe **26** exhibits a 68-fold fluorescence enhancement at 532 nm at 25 °C in PBS solution (20 mM, pH 7.4) containing 10% CH_3_CN. The probe shows a low detection limit of 2.46 μM, low cytotoxicity, and good selectivity for H_2_S. Time-dependent fluorescence measurements indicate that the maximum fluorescence intensity is reached within 40 min after the addition of Na_2_S. Probe **26** was further applied for the fluorescence detection of H_2_S in HeLa cells [113].

### 3.3. Detecting H_2_S by Other Reactions

Although reduction and nucleophilic aromatic substitution (S_N_Ar) reactions are the most widely employed strategies for designing fluorescent probes for H_2_S detection, several other reaction mechanisms have also been utilized. These alternative approaches include intramolecular cyclization, Michael addition, nucleophilic addition, and displacement of metal ions triggered by H_2_S. Such reaction pathways exploit the strong nucleophilicity and acidity of H_2_S under physiological conditions. By incorporating appropriate reactive sites into fluorophores, these mechanisms can generate measurable fluorescence changes after reaction with H_2_S. These strategies expand the structural diversity of H_2_S-responsive probes and provide additional design options for selective sensing.

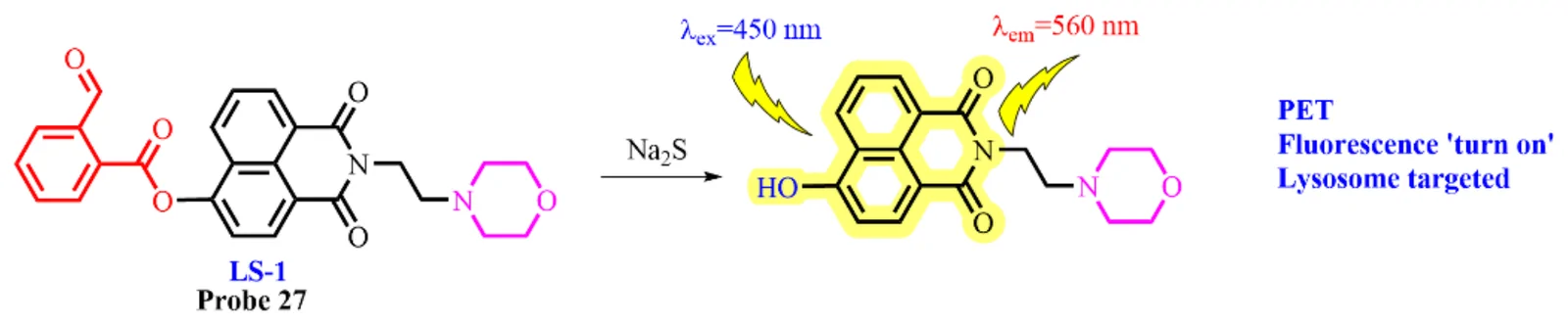



In 2018, Velusamy et al. reported probe **27**, a lysosome-targeted self-immolative fluorescent probe for detecting H_2_S. In this design, a 2-formylbenzoate moiety was appended to a 1,8-naphthalimide fluorophore as the recognition unit. Self-immolative reactions have been widely employed in the design of H_2_S fluorescent probes, in which H_2_S initiates a spontaneous cascade reaction that ultimately releases or activates the fluorophore, resulting in a pronounced fluorescence turn-on response. This strategy offers the advantages of high signal amplification and low background fluorescence, making it attractive for sensitive H_2_S detection. In the presence of Na_2_S, the aldehyde group reacts with H_2_S to form a thiohemiacetal intermediate, which subsequently undergoes intramolecular cyclization with the adjacent ester group, leading to the release of 4-hydroxy-1,8-naphthalimide as the fluorescent product. As a result, probe **27** exhibits absorption and emission maxima at 450 nm and 560 nm, respectively, with a significant fluorescence enhancement upon exposure to Na_2_S. The probe shows high sensitivity and selectivity toward H_2_S over other biological analytes. In addition, probe **27** displays low cytotoxicity and good stability over the physiological pH range. Probe **27** was further applied to detect intracellular endogenous H_2_S in HT29 cells with lysosomal localization [114].

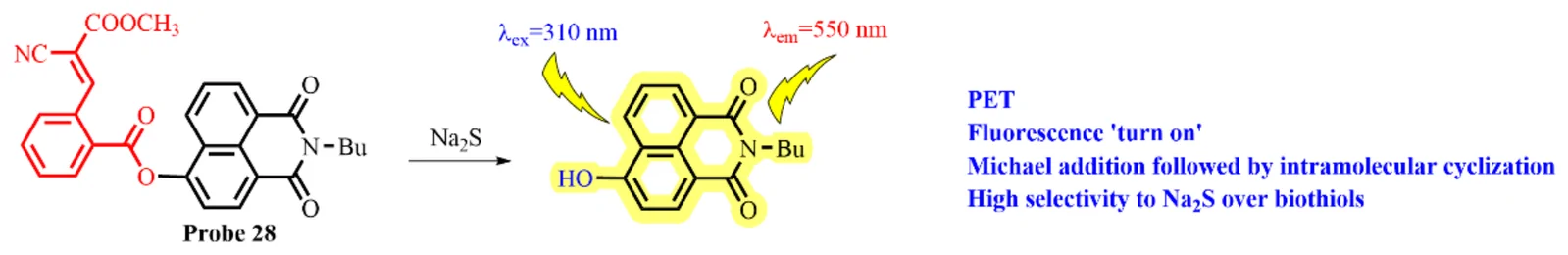



Gao et al. reported probe **28**, which employs a Michael addition followed by intramolecular cyclization as the sensing mechanism for H_2_S detection. In the presence of Na_2_S, probe **28** exhibits a significant fluorescence enhancement at 550 nm in MeCN/PBS buffer (1:1, 50 mM PBS), with an approximately 75-fold increase in fluorescence intensity. A good linear correlation (R = 0.98) was observed between the fluorescence intensity at 550 nm and the H_2_S concentration in the range of 0–20 μM. In addition, a strong fluorescence enhancement at 550 nm was observed over a wide pH range of 4–8 in the presence of H_2_S. Kinetic studies indicated that the reaction between probe **28** and NaHS was completed within 5 min in PBS buffer at room temperature [115].

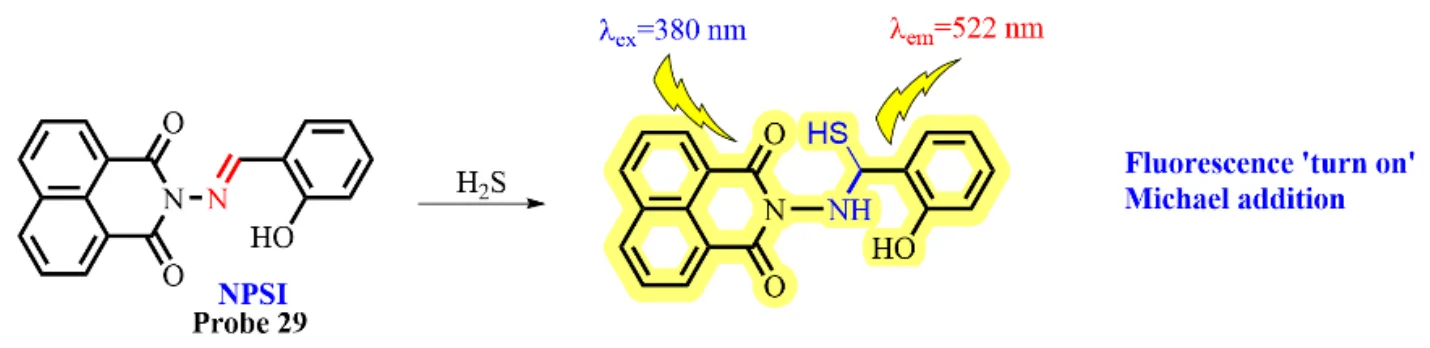



Kundu et al. reported probe **29** for detecting H_2_S in the gas phase, with potential applications in the petroleum industry. Probe **29** utilizes a nucleophilic addition reaction as the sensing mechanism, in which H_2_S reacts with a C=N bond in the *N*-imide moiety of 1,8-naphthalimide. In the presence of H_2_S, a significant fluorescence enhancement at 522 nm was observed, accompanied by a distinct color change. Probe **29** was further applied for the detection of H_2_S in crude petroleum oil [116].

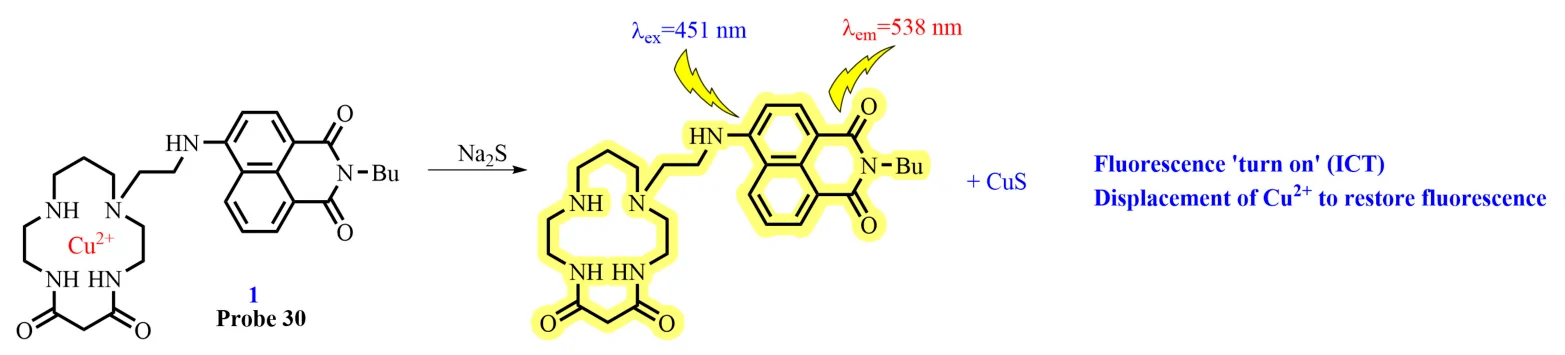



Probe **30** was designed as an anion probe for detecting S^2−^. The probe consists of a 1,8-naphthalimide fluorophore appended with a nitrogen-containing crown ether as the metal-binding unit. In the absence of Cu^2+^, probe **30** exhibited a strong fluorescence emission at 538 nm. Upon addition of Cu^2+^, coordination of the metal ion with the crown ether moiety resulted in nearly complete fluorescence quenching. When S^2−^ was introduced in H_2_O/CH_3_OH (3:1, *v*/*v*, Britton–Robinson buffer, pH 7.1), it reacted with Cu^2+^ to form a stable copper sulfide species, thereby restoring the fluorescence of the probe via ICT. Probe **30** shows high selectivity toward S^2−^ over other anions [117].

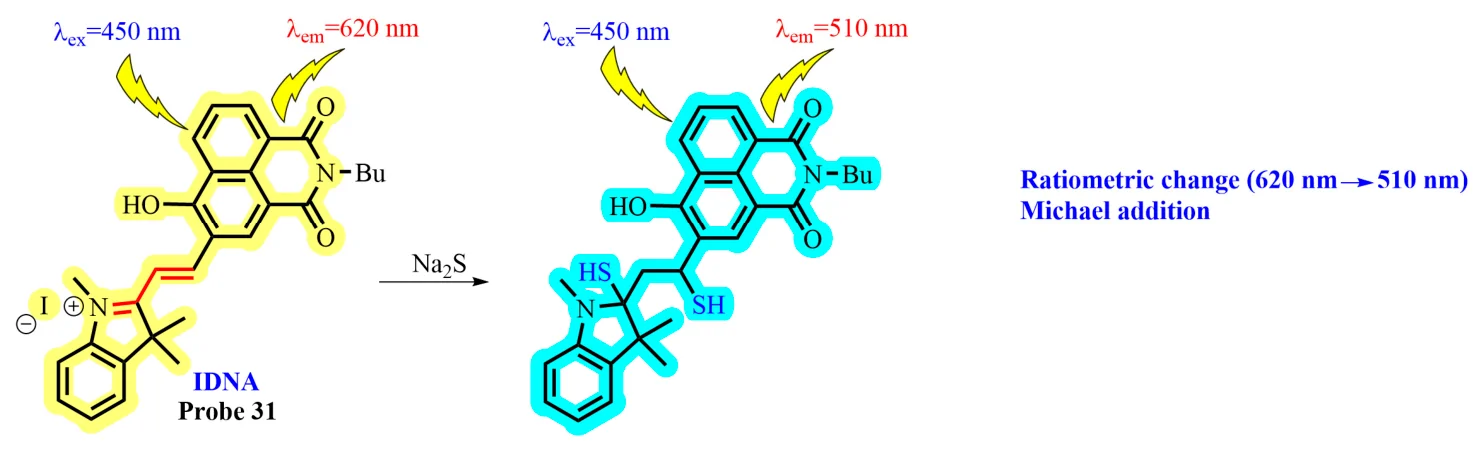



Probe **31** was constructed by appending an indole group to 4-hydroxy-1,8-naphthalimide, forming an extended conjugated system. This structure exhibits fluorescence emission at 620 nm in DMSO/H_2_O (4:6, *v*/*v*) solution. In the presence of Na_2_S, the thiol species was added to the C=C and C=N bonds through a nucleophilic addition reaction, which disrupted the conjugated system of probe **31**. As a result, a new emission band at 510 nm appeared, generating a ratiometric fluorescence response. Probe **31** showed a good linear relationship between fluorescence intensity and H_2_S concentration in the range of 0.3–0.47 mM, with a detection limit of 7 μM. The probe was applied for fluorescence detection of H_2_S in HeLa cells. Although probe **31** lacks a specific mitochondrial-targeting group, mitochondrial localization was suggested based on co-localization fluorescence imaging experiments [118].

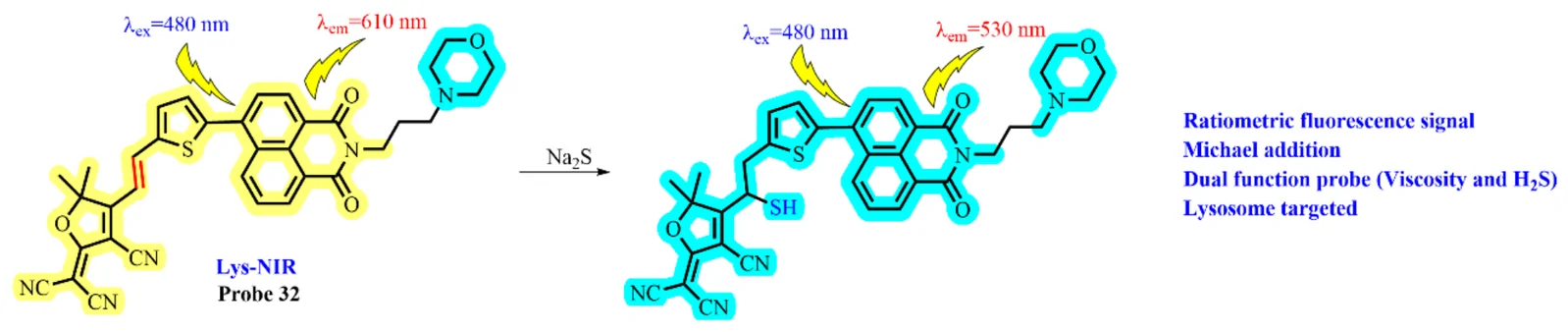



In 2026, Li et al. reported probe **32** as lysosome-targeted near-IR probe for detecting H_2_S. Probe **32** was constructed as an extended conjugation at C4 of 1,8-naphthalimide including a C=C bond, which can react with Na_2_S via Michael addition reaction, which interrupts the conjugation and induces a blue shift in the fluorescence emission from 610 nm to 530 nm in the MeOH/HEPES (3:7, *v*/*v*) system. Moreover, the C=C in the conjugation was also sensitive to the viscosity of media, showing a fluorescence enhancement at 640 nm. Therefore, probe **32** can be used as a dual-function probe for detecting H_2_S and viscosity. Probe **32** has been applied for H_2_S detection in different environments, including water/beer, food spoilage monitoring, and living cells [119].

## 4. Challenges and Perspectives

Although 1,8-naphthalimide has been widely used for fluorescent probe design, several challenges remain in its application to H_2_S detection. The intrinsic photophysical properties that make 1,8-naphthalimide attractive for probe design can also introduce experimental complexity. For example, the emission wavelength and intensity of 1,8-naphthalimide derivatives can be strongly influenced by pH, solvent polarity, and other environmental factors. Even relatively small changes in experimental conditions may therefore produce significant changes in fluorescence response and introduce potential interference. In addition, many 1,8-naphthalimide derivatives have limited aqueous solubility and require organic cosolvents, which can complicate their use in biological systems by affecting probe behavior and cellular responses. Furthermore, multiple photophysical pathways, such as PET and ICT, may operate simultaneously or compete with each other. This overlap can complicate the interpretation of fluorescence changes and, in some cases, make the proposed sensing mechanism difficult to establish.

The use of different H_2_S sources to evaluate fluorescent probes is another important challenge. Na_2_S, NaSH, and H_2_S solutions have all been used for H_2_S probe evaluation, but these sources differ in stability, speciation, and handling. NaHS is hygroscopic and can form hydrates, which may change its composition over time, while H_2_S solutions are volatile and may gradually lose H_2_S during storage and experiments. The use of Na_2_S can also affect the kinetics of the sensing process because S^2−^ undergoes hydrolysis under near-neutral aqueous conditions and generates HS^−^. Therefore, the use of different H_2_S sources can lead to inconsistencies in notation and make it difficult to directly compare results from different studies. More consistent methods for preparing, quantifying, and reporting H_2_S sources would improve the reproducibility and comparability of H_2_S sensing studies.

Selectivity in biological environments represents another important challenge. Biologically relevant thiols, particularly glutathione (GSH) and cysteine (Cys), are present at relatively high concentrations and can interfere with H_2_S detection for reaction-based fluorescent probes. However, the extent of interference from GSH and Cys varies substantially among different probes. For example, some DNBS-based probes have been reported to show higher selectivity toward Cys than HS^−^. Such differences in reported selectivity may limit the reliability of H_2_S detection in complex biological environments.

The reporting of the limit of detection (LOD) is another important issue that deserves greater attention. LOD is an important parameter for evaluating fluorescent H_2_S probes, particularly when low-concentration detection is claimed. However, different approaches and experimental conditions have been used to determine LOD, and some reported values may reflect instrument noise rather than the experimentally measurable detection limit. In other cases, a statistically calculated LOD may be reported without demonstrating the actual detection capability of the probe. More consistent methods for determining and reporting LOD, together with clear descriptions of the experimental conditions, would allow more meaningful comparison of sensing performance among different probes.

One particularly interesting feature of H_2_S fluorescent probes is that their recognition mechanisms are largely based on organic reactions. This characteristic has attracted considerable interest from organic chemists and provides opportunities to develop new reaction-based recognition units. The development of new H_2_S-triggered reactions, together with new fluorophores, may provide additional strategies to improve the sensitivity and selectivity of H_2_S probes.

Continuous monitoring of H_2_S in biological systems is another important direction. Most currently reported H_2_S probes rely on irreversible reactions, in which the probe is consumed after reacting with H_2_S and therefore provides only a one-time measurement. Developing probes based on reversible recognition mechanisms could enable continuous monitoring of H_2_S concentration and provide more useful information about its dynamic changes in biological systems. Such approaches may further expand the biological and potential medical applications of H_2_S sensing.

Although many H_2_S probes have been evaluated in biological systems, their applications have mainly focused on fluorescence imaging in cultured cells and animal models, particularly HeLa cells and zebrafish. Extending H_2_S detection to more complex biological samples, such as serum, plasma, and tissue samples, could provide a closer connection between probe development and biomedical applications. Such studies would also provide an important test of probe selectivity and performance in complex biological environments.

Finally, despite extensive studies on the photophysical properties of 1,8-naphthalimide and its numerous applications in fluorescence sensing, some structure–property and structure–function relationships remain to be established. Even minor structural modifications can sometimes lead to significant changes in photophysical properties and sensing behavior, making the systematic investigation of these relationships challenging. Further systematic studies linking molecular structure, photophysical properties, reaction behavior, and sensing performance could provide useful guidelines for the rational design of 1,8-naphthalimide-based H_2_S probes. Integrating advances in photochemistry, organic chemistry, biochemistry, chemical biology, medicinal chemistry, and molecular biology may facilitate the rational design of more reliable, selective, and functionally versatile H_2_S fluorescent probes, ultimately advancing their applications in cellular imaging, disease diagnosis, and biomedical research.

## 5. Conclusions

**Figure 4:**
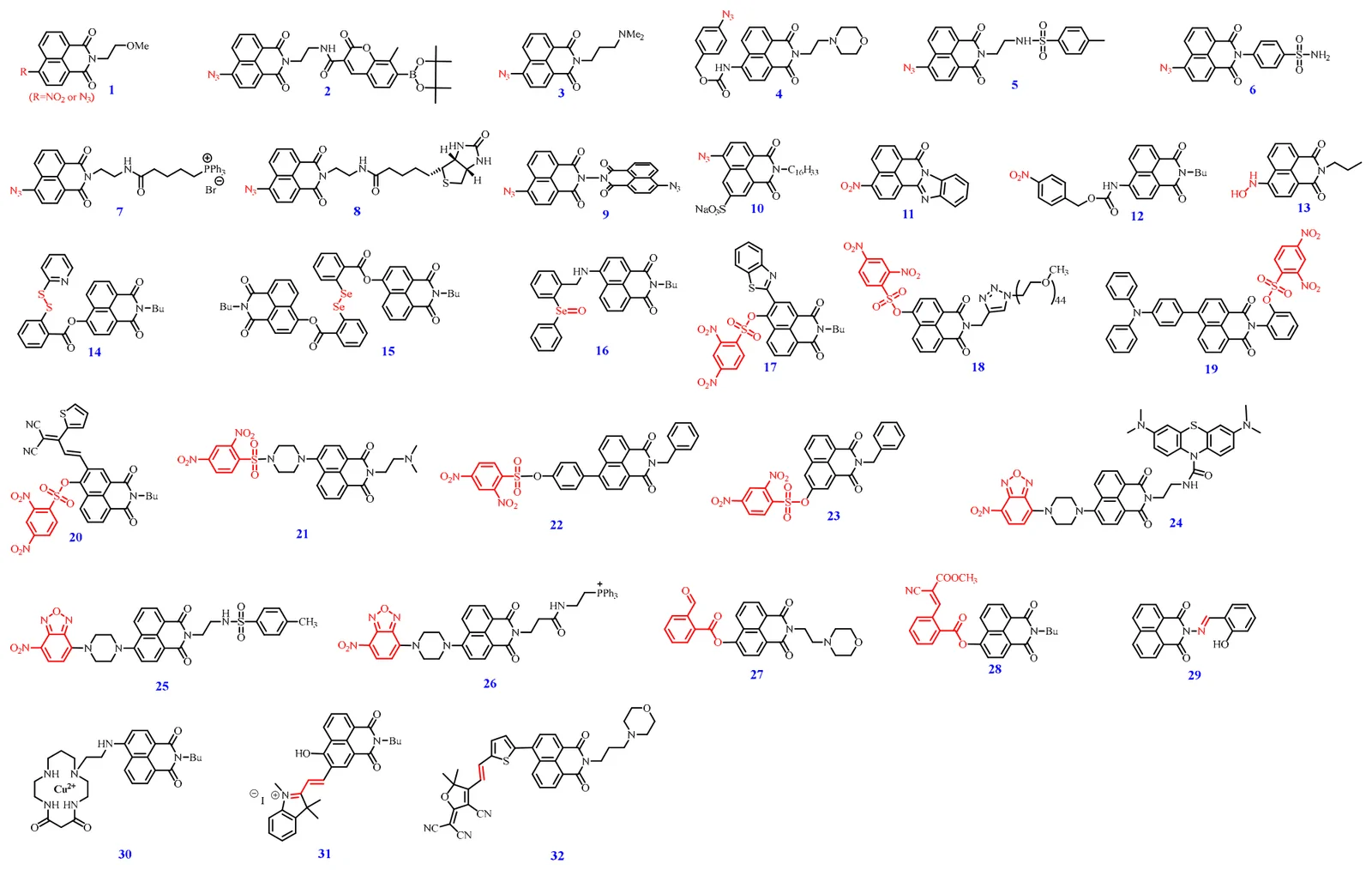
Reaction-based fluorescent probes (1–32) for detecting H_2_S.

The 1,8-naphthalimide scaffold has been extensively employed as a fluorophore in the development of fluorescent probes for H_2_S detection, owing to its distinctive photophysical properties and high sensitivity to structural changes. Taking advantage of the strong nucleophilicity and reducing ability of H_2_S, a variety of reaction-based sensing strategies have been developed using the 1,8-naphthalimide scaffold, enabling sensitive and selective H_2_S detection in diverse environments. In this review, we have summarized 32 fluorescent probes based on diverse sensing and recognition mechanisms, with particular emphasis on their sensing principles and applications (Figure 4 and Table 1). Collectively, these studies demonstrate the versatility of the 1,8-naphthalimide scaffold for the development of fluorescent probes for H_2_S detection, particularly in biological sensing applications. Building on these advances, further investigation into the structure–function relationships of 1,8-naphthalimide scaffold are expected to facilitate the rational design of fluorescent probes with enhanced sensitivity, selectivity, and overall performance for H_2_S detection in complex biological environments.

## Figures and Tables

**Table 1:** Summary features of probe 1–32.

Probe	Reaction Used	Sensing Mechanism	Feature	Reference
1	Reduction of azide	ICT	First H_2_S probe using reduction based on 1,8-naphthalimide scaffold	[89]
2	Reduction of azide	ICT/FRET	Lysosome-targeted	[90]
3	Reduction of azide	ICT	Lysosome-targeted	[91]
4	Reduction of azide	ICT	Two-photon excitation	[92]
5	Reduction of azide	ICT	Endoplasmic reticulum-targeted	[93]
6	Reduction of azide	ICT	Golgi-targeted	[94]
7	Reduction of azide	ICT	Mitochondria-targeted	[95]
8	Reduction of azide	ICT	Cancer cell–targeted, two-photon excitation	[96]
9	Reduction of azide	ICT	Monitoring of H_2_S-induced DNA damage	[97]
10	Reduction of azide	ICT	Cell surface-targeted	[98]
11	Reduction of nitro	ICT	Fast response time	[99]
12	Reduction of nitro	ICT	Reduction followed by intramolecular cyclization	[100]
13	Reduction of hydroxylamine	ICT	H_2_S-triggered reduction of hydroxylamine	[99]
14	Reduction of disulfide	ICT	Reduction followed by intramolecular cyclization	[100]
15	Reduction of diselenide	ICT	Increased selectivity to H_2_S over biothiols	[101]
16	Reduction of selenoxide	ICT	Dual-function probe for H_2_S and HOCl	[102]
17	S_N_Ar (DNBS)	PET/ICT	For detecting H_2_S and biothiols	[105]
18	S_N_Ar (DNBS)	PET/ICT	Fabricated into test paper strips	[106]
19	S_N_Ar (DNBS)	PET/AIE/TICT	PET from N-imide, Application using paper strip	[107]
20	S_N_Ar (DNBS)	PET	Near-IR emission, Dual function (H_2_S and viscosity)	[108]
21	S_N_Ar (DNBS)	PET	Probe based on sulfonyl imide structure	[109]
22	S_N_Ar (DNBS)	ICT	Ratiometric signal	[110]
23	S_N_Ar (DNBS)	PET/ESPT	C3 on 1,8-naphthalimide, red-shift emission	[85]
24	S_N_Ar (NBD)	PET	Dual-function probe	[111]
25	S_N_Ar (NBD)	PET	ER-targeted	[112]
26	S_N_Ar (NBD)	PET	Mitochondria-targeted	[113]
27	Intramolecular cyclization	ICT	lysosome-targeted	[114]
28	Michael addition	ICT	Michael addition	[115]
29	Addition	PET	Detecting H_2_S in gas phase	[116]
30	Displacement	ICT	Detecting S^2−^	[117]
31	Nucleophilic addition	ICT	Ratiometric signal, Mitochondria-targeted	[118]
32	Michael addition	ICT	lysosome-targeted, NIR emission	[119]

## Data Availability

No new data were created or analyzed in this study.
